# Sex Hormone Estrone Sulfate Serendipitously Downregulates Intercellular Tunneling Nanotube Conduits, EMT, and Migration in Breast and Pancreatic Cancer Cells

**DOI:** 10.3390/cancers18203354

**Published:** 2026-10-16

**Authors:** Izzah Wahab, Yunfan Li, Myron R. Szewczuk

**Affiliations:** Department of Biomedical & Molecular Sciences, Queen’s University, Kingston, ON K7L 3N6, Canada; 19iw4@queensu.ca (I.W.); 18yl210@queensu.ca (Y.L.)

**Keywords:** estrone, MDA-MB-231, MCF-7, PANC-1 and RawBlue cells, epithelial-to-mesenchymal (EMT) transition, a tunneling nanotube assay, scratch wound

## Abstract

**Aim:** Estrone (E1) is of particular interest as most breast cancers are diagnosed after menopause, the period in which E1 becomes the dominant estrogen. Triple-negative breast cancer (TNBC) is a highly invasive cancer that lacks hormone receptors for estrogen (ER), progesterone (PR), and HER2 (human epidermal growth factor receptor-2). TNBC management remains clinically challenging despite recent advances in targeted and immunotherapeutic approaches. This study investigated the effects of the estrogen hormone estrone sulfate (E1S) on cancer cells. Here, E1S engages in non-classical biased G protein-coupled estrogen receptor (GPER) signaling in TNBC MDA-MB-231 cells, estrogen receptor-positive MCF-7 breast cancer cells, and pancreatic PANC-1 cancer cells, which express estrogen, progesterone, and androgen receptors. **Methods:** We performed sialidase and colocalization assays in breast MDA-MB-231 and MCF-7 cells and pancreatic PANC-1 cancer cells. An epithelial-to-mesenchymal (EMT) transition, a tunneling nanotube assay, and a scratch wound assay were conducted to elucidate the cellular behaviors of E1S. We used a RawBlue reporter macrophage cell line to measure NFκB activity using the secreted embryonic alkaline phosphatase (SEAP) assay, and we measured cellular viability and metabolic activity using the AlamarBlue assay. **Results:** E1S induced NMBR/MMP9/Neu1 signaling in MDA-MB-231, MCF-7, PANC-1 and RawBlue cells. E1S increased E-cadherin expression, reduced N-cadherin and vimentin expression, decreased tunneling nanotube density, and reduced scratch wound migration. E1S increased SEAP activity, suggestive of increased nuclear factor-κB (NF-κB) and activator protein-1 (AP-1)-mediated transcription. E1S treatment did not affect cell viability and metabolic activity in triple-negative MDA-MB-231 breast cells using the AlamarBlue assay in a dose-dependent manner. **Conclusions:** E1S binding to GPER potentiates a biased heterodimerization with the GPCR neuromedin B receptor (NMBR) to activate matrix metalloproteinase 9 (MMP9) and neuraminidase 1 (Neu1), a signaling paradigm with previously established receptor tyrosine kinase (RTK) and Toll-like (TLR) receptors. Biased, temporal GPER signaling has significant implications for breast and pancreatic cancer cellular behavior, which may be leveraged to inform therapeutic strategies for aggressive forms of breast and pancreatic cancers. This research study is mechanistic in nature and may have potential translational implications.

## Simple Summary

Our findings unveil a novel role of the sex hormone estrone (E1) in progression of triple-negative breast, breast, and pancreatic cancers. Here, estrone sulfate (E1S) serendipitously downregulates intercellular tunneling nanotube conduits, epithelial-to-mesenchymal (EMT) transition, and migration in TNBC MDA-MB-231 cells, estrogen receptor-positive MCF-7 breast cancer cells, and pancreatic PANC-1 cancer cells. These findings reveal a novel signaling model in which E1S-mediated partial or weak agonism at the G protein-coupled estrogen receptors (GPERs) results in a biased response, involving the GPCR NMBR in activating the MMP9/Neu1 signaling axis of receptor tyrosine kinases (RTKs), thereby altering cancer cell progression. This GPER-biased NMBR heterodimerization signaling paradigm may be leveraged to treat breast and pancreatic cancers, wherein ligands of varying strengths, such as E1S, and their downstream signaling mechanism(s) can be investigated and exploited to mitigate cancer progression.

## 1. Introduction

Breast cancers are hormone-dependent diseases [1,2]. Nearly 70–80% of breast cancers express ERs and/or PRs [1,2]. This association reflects the role of estrogens in altering the breast architecture and development [3]. For example, the rudimentary mammary structure formed during the prenatal period transforms into the mature mammary gland after puberty in females [3]. This transformation, characterized by ductal development, fat deposition, and connective tissue growth, is largely orchestrated by estrogens. Growth hormone (GH) and insulin-like growth factor-1 (IGF-1) also play significant but comparatively auxiliary roles [1,3]. Notably, alterations of the breast structure by estrogen are not exclusively benign; in fact, clinical and experimental data show that estrogen is integral to the proliferation and progression of breast cancer [1,2]. In various malignant breast cells, the ER signaling pathway promotes proliferative and pro-survival signals. It inhibits apoptosis by upregulating Bcl-2, an anti-apoptotic proto-oncogene [1].

However, the role of estrogen in cancers lacking ERs and PRs is limited and inconsistent [4,5,6,7,8]. Therefore, given the significant role of estrogens in breast development and cancer, it is imperative to distinguish the unique biological properties of endogenous estrogens and to analyze how these estrogens affect cancers that do not rely on classical estrogen signaling pathways. The term estrogen refers to a class of sex hormones primarily involved in the development and regulation of the female reproductive system [9,10]. Estrogens are ubiquitous and therefore impact nearly all physiological systems and tissues, including the neuroendocrine, skeletal, immune, and cardiovascular systems [9,10,11]. Four endogenous forms of estrogen exist, namely estrone (E1), estradiol (E2), estriol (E3), and estetrol (E4), which vary in potency and abundance throughout a female’s lifetime [9,11,12]. Differences in potency reflect variations in agonist affinity for cognate receptors and intrinsic efficacy, which together influence the magnitude and nature of downstream signaling responses [9,11,12]. From puberty to menopause, E2 is the predominant estrogen hormone. It is widely recognized as the most potent estrogen due to its high affinity for ERs [12]. Conversely, E1 dominates during the postmenopausal period [12]. E3 has intermediate potency and predominates during pregnancy [12]. The least potent estrogen, E4, is regarded as the “estrogen of fetal life”, as the fetal liver synthesizes it and it is solely present during pregnancy [11]. As such, E4 levels are relatively high in the fetus but lower in maternal circulation [11].

Of the various forms of estrogen, E1 is of particular interest as most breast cancers are diagnosed after menopause, the period in which E1 becomes the dominant estrogen [13]. However, E1 is estimated to have less than 10% the relative potency of E2 in conventional ligand-binding assays and in vitro bioassays; its circulating serum concentrations may be 100-fold greater than those of E2 in postmenopausal women [14,15]. Triple-negative breast cancer (TNBC), however, is most frequently diagnosed in premenopausal women, a demographic in which E2 predominates [16]. Therefore, due to its high potency and relevance in the premenopausal window, the effects of E2 have been more extensively characterized than those of E1 [14,15]. Because E2 has been extensively studied, analyzing the effects of E1 in its abundant storage form on TNBC or other cancers is necessary to address gaps in the literature. Examining the role of E1 may elucidate potential non-classical signaling pathways independent of ERs, PRs, and HER2 that may influence cancer development and progression. Importantly, this use of non-classical estrogen signaling is not limited to the postmenopausal period and may be broadly applicable across age groups.

In postmenopausal women, this marked reduction in serum estrogen levels results from the cessation of ovarian E2 production [17]. Consequently, estrogen synthesis primarily occurs in peripheral adipose tissue as E1 [17]. In adipose tissue and, to a lesser degree, in bone, breast, and brain, E1 is produced by the aromatization of adrenal androstenedione [13,17]. Notably, while ovarian-synthesized estrogen is largely released into the bloodstream, estrogen produced in extragonadal sites has localized effects, acting as a paracrine and/or intracrine factor to maintain tissue-specific functions [12].

Studies have shown that 2-hydroxyestrone and 2-hydroxyestradiol inhibit cell growth and proliferation [18]. Thus, some researchers have coined 2-hydroxyestrone a “good estrogen” [19]. Estrogen sulfation is noteworthy because it facilitates estrogen storage in breast tissue [12,20]. Estrogen sulfotransferase (SULT1E1) catalyzes the sulfation of E1 and E2 efficiently [21]. Estrogen sulfatase (STS) mediates the reverse desulfation reaction, reactivating estrogens [21]. Estrone sulfate (E1S) is the most abundant circulating estrogen in adults and serves as an inactive reservoir that provides E1 and E2 [22,23]. E1 can be reduced to E2 by 17β-hydroxysteroid dehydrogenase 1 (HSD17B1), whereas the reverse oxidation of E2 to E1 is mediated by 17β-hydroxysteroid dehydrogenase 2 (HSD17B2) [24].

In summary, in premenopausal women, the ovaries are the primary site of estrogen production [24]. Conversely, in postmenopausal women, ovarian production of estrogen ceases [24]. As such, the most potent estrogen, E2, declines in serum and no longer overshadows E1S, the primary circulating steroid throughout adulthood [15,25,26]. After this estrogenic shift, the main source of active estrogen in any tissue is local conversion [24]. In the breast, SULT1E1 and 17β-hydroxysteroid dehydrogenases facilitate conversion [24]. Correspondingly, enzyme expression levels and substrate availability determine the balance and overall effect of sex steroids in local tissues [24].

Estrogens act through two types of receptors: (i) classical nuclear receptors and (ii) cell surface membrane receptors [12,27,28]. Classical nuclear receptors include estrogen receptor α (ERα) and estrogen receptor β (ERβ) [12,27,28]. Cell-membrane receptors include the G protein-coupled estrogen receptor (GPER) [12,27,28].

ERα is predominantly expressed in the gonadal organs, including the breast, uterus, ovary, prostate, and testes [12,27,28]. However, the receptor is also expressed at lower levels in bone, kidney, liver, adipose tissue, and brain [12,27,28]. Notably, TNBCs do not express ERα but may express variable levels of ERβ and often overexpress the human epidermal growth factor receptor (EGFR), even though they do not express HER2 [4,16,29,30,31]. ERβ is more commonly expressed in non-gonadal organs, including the colon, bone marrow, lungs, bladder, brain, and vascular endothelium [12,27,28]. In contrast, GPERs are present in several tissues throughout the body, including but not limited to the breasts, brain, uterus, ovaries, vascular system, kidneys, and the central and peripheral nervous systems [12,27,28,32]. Torres-López et al. [33] provided an excellent review on the GPER structure, ligand diversity, and their induced GPER signaling pathways, cancer progression, and mechanism(s) of chemoresistance. Notably, they also focused on GPER involvement in different cancer types and GPER’s role in the tumor microenvironment [33].

ER-dependent signaling can be categorized into ligand-dependent or ligand-independent, and as genomic or non-genomic, depending on how signaling is initiated and the resulting cellular event (e.g., altered gene expression or the initiation of signaling cascades) [12,27,28]. In the classical, direct pathway, estrogen binds to ERs [12,27,28]. As a result, the estrogen–ER homo- or heterodimeric complex translocates to the nucleus. It binds to estrogen response elements (EREs) located in or near target gene promoters to modulate gene expression [12,27,28].

Estrogen biology is multifaceted and employs signaling mechanisms beyond classical ER-dependent signaling, particularly in cancers such as TNBC that lack ERα and variably express ERβ [4,16,29]. Nevertheless, TNBC still responds to estrogenic cues [4,16,29]. Therefore, conceptualizing how E1S, the widely abundant storage form of E1, engages in non-classical signaling pathways to influence cancer progression is essential [4,16,29]. Consequently, defining these pathways may advance our understanding of estrogen-driven signaling in cancer cells and reveal new therapeutic opportunities.

Taken together, the findings of the present study support a novel signaling model in which E1S-mediated partial or weak agonism at GPERs results in biased responses involving the NMBR/MMP9/Neu1 signaling axis of RTK receptors, thereby altering TNBC and other cancer progression. GPCRs can operate within multi-receptor complexes that include other GPCRs, RTKs, MMPs, and Neu1 [34]. The prior documented involvement of NMBR/MMP9/Neu1 signaling, particularly through receptor desialylation and activation, provides a mechanistic framework through which GPER signaling may alter cellular outcomes in a biased manner [34]. In the proposed signaling model, E1S interacts with GPERs to form a heteromeric complex with NMBR, thereby activating MMP9-Neu1 cross-talk and subsequent RTK and TLR downstream signaling.

## 2. Materials and Methods

### 2.1. Cell Lines

The MDA-MB-231 (ATCC^®^ HTB-26™, Manassas, VA, USA) cell line was used. MDA-MB-231 is a TNBC cell line obtained from a pleural effusion of a 51-year-old Caucasian female with metastatic mammary adenocarcinoma [35,36]. MCF-7 is a human, epithelial morphology, estrogen- and progesterone-receptor-positive breast cancer cell line (ATCC HTB-22TM, Manassas, VA, USA). PANC-1 (human, epithelial morphology, ATCC CRL-1469TM, Manassas, VA, USA) is isolated from pancreatic duct epithelioid carcinoma. We also used the RAW-BlueTM cell line (InvivoGen, San Diego, CA, USA) to assess NF-κB-dependent signaling. The RAW-Blue^TM^ cell line is a mouse macrophage reporter cell line derived from murine RAW 264.7 macrophages that stably expresses an optimized secreted embryonic alkaline phosphatase (SEAP) reporter gene inducible by NF-κB and AP-1 [37]. To ensure appropriate gene expression, RAW-Blue^TM^ cells were cultured in a medium containing Zeocin as the selectable marker.

Cell lines were cultured in Dulbecco’s Modified Eagle Medium (DMEM; Gibco, Rockville, MD, USA) with the addition of 10% fetal bovine serum (FBS; HyClone, Logan, UT, USA) and 5 μg/mL Plasmocin^TM^ (InvivoGen, San Diego, CA, USA) to reduce the risk of contamination by mycoplasma. All cells were cultured in T-75 tissue culture flasks at 37 °C in a humidified incubator with 5% CO_2_. At approximately 70–80% confluency, cells were passaged at least 5 times after thawing to allow recovery from cryopreservation and stabilize growth and signaling parameters before experimental use.

### 2.2. Reagents

Estrone-3-sulfate sodium salt (E1S) was purchased from MedChemExpress (Monmouth Junction, NJ, USA). E1S powder was dissolved in phosphate-buffered saline (PBS) to prepare a 5 mg/mL stock solution, which was aliquoted and stored at −20 °C. Immediately before treatment, we diluted stock solutions in PBS or serum-starved medium to the desired concentration. GPCR NMBR was antagonized with the specific antagonist BIM-23127 at 30 nM (Tocris Bioscience, Bristol, UK). MMP9 activity was inhibited using a selective MMP9 inhibitor (MMP9i; 2 μM; GLPBIO Technology, Montclair, CA, USA). Neu1 activity was inhibited using oseltamivir phosphate (OP; 300 μg/mL; ≥99% purity, Batch No. MBAS20014A; Active Pharma Sciences Ltd., New Mangalore, Karnataka, India).

### 2.3. Sialidase Assay

MDA-MB-231 cells were seeded onto 12 mm circular glass coverslips in sterile 24-well tissue culture plates (Falcon; Becton, Dickinson & Company, Mississauga, ON, Canada). Cells were maintained in conditioned medium and incubated at 37 °C in a humidified atmosphere containing 5% CO_2_ until ~70–80% confluence, as optimized for the live cell sialidase assay [36]. After removing the medium, cells were washed with PBS and incubated in serum-free DMEM for 60 min to minimize the effects of exogenous factors in FBS. Subsequently, 4 μL of the fluorogenic sialidase substrate, 2-(4-methylumbelliferyl)-α-D-N-acetylneuraminic acid (4-MUNANA; 0.318 mM; Biosynth International Inc., Itasca, IL, USA), diluted in Tris-buffered saline (TBS), was added to control samples. For treated samples, we added all compounds to a final volume of 4 μL. Accordingly, we co-applied 4-MUNANA with either EGF (30 ng/mL) or E1S (0.1–50 nM). We conducted subsequent assays in the presence of pathway-specific inhibitors. We antagonized NMBR with BIM-23127 (30 nM; Tocris Bioscience, Bristol, UK). MMP9 activity was inhibited using a selective MMP9 inhibitor (MMP9i; 2 μM; GLPBIO Technology, Montclair, CA, USA). Neu1 activity was inhibited using oseltamivir phosphate (OP; 300 μg/mL; ≥99% purity, Batch No. MBAS20014A; Active Pharma Sciences Ltd., New Mangalore, Karnataka, India). After adding all relevant compounds, we inverted coverslips bearing adherent cells onto microscope slides.

Fluorescence was detected using excitation and emission wavelengths of 365–380 nm and 445–454 nm, respectively, with a Zeiss Imager M2 epifluorescent microscope equipped with a 10× objective to capture fluorescent and phase-contrast images (Carl Zeiss Canada Ltd., Toronto, ON, Canada). We captured all images about 1 min after treatment with 4-MUNANA and E1S alone or with inhibitors. Mean fluorescence intensity was quantified from 50 peripheral regions per cell across three images per slide (*n* = 3–4) using ImageJ software (version 1.5g, Java 1.8.0_345, 64-bit). Each experiment was performed in triplicate.

### 2.4. Colocalization Assay

Cells were seeded onto 12 mm circular glass coverslips placed in sterile flat-bottom 24-well tissue culture plates (Falcon; Becton, Dickinson & Company, Mississauga, ON, Canada). Cells were grown in complete growth medium and incubated at 37 °C in a humidified atmosphere with 5% CO_2_ until they reached ~70–80% confluence. Subsequently, the cells were serum-starved for 24 h, fixed using 4% paraformaldehyde (PFA) for 20 min, washed, and permeabilized with 0.2% Triton X-100 for 5 min at room temperature. Cells were then blocked with 4% bovine serum albumin (BSA) in 0.05% Tween-20 in Tris-buffered saline (TBST) solution for 24 h at 4–5 °C.

The fixed cells were incubated with rabbit recombinant monoclonal anti-G protein-coupled receptor-30 conjugated with Alexa Fluor^®^ 594 antibody (ab311828; Abcam Inc. 152 Grove Street, Suite 1100, Waltham, MA, USA) and a mouse monoclonal IgG anti-Neu-1 Alexa FluorTM 488-conjugated antibody (sc-166824, Santa Cruz Technologies, Dallas, TX, USA) at 1:20 dilution, with gentle rocking at 4–5 °C for 24 h.

The coverslips with the stained adherent cells were then mounted onto microscope slides on 3 μL of VECTASHIELD DAPI mounting medium (DAPI; VECTH1500, MJS BioLynx Inc., Brockville, ON, Canada). Images were then captured using a Zeiss M2 epifluorescent microscope (40× objective magnification, 488 nm and 594 nm excitation wavelengths; Carl Zeiss Canada Ltd., Toronto, ON, Canada). The Pearson correlation coefficient, which demonstrates colocalization between the two fluorescent-antibody channels, was determined using AxioVision software, Rel. 4.6. A correlation value in the range of 0.5–1.0 indicates a significant positive relationship.

### 2.5. EMT Assay

Cells were seeded onto 12 mm circular glass coverslips placed in sterile 24-well tissue culture plates (Falcon; Becton, Dickinson & Company, Mississauga, ON, Canada). Cells were grown in complete growth medium and incubated at 37 °C in a humidified atmosphere containing 5% CO_2_ until ~70–80% confluence was reached. Subsequently, control samples received 300 μL of serum-free medium, while treated cells received 300 μL of E1S (10 nM) diluted in serum-free medium for 24 h. Cells were washed with 1× PBS and fixed with 4% PFA for 20 min at room temperature. Cells were then washed and permeabilized with 0.2% Triton X-100 for 5 min at 37 °C. After incubation, the cells were washed and blocked with 4% BSA in 0.05% TBST for 20 min at room temperature. Following washing and blocking, cells were incubated with antibodies targeting EMT-associated markers, including anti-E-cadherin (Alexa Fluor 594), anti-N-cadherin (Alexa Fluor 594), or anti-vimentin (Alexa Fluor 594) (Santa Cruz Biotechnology, Dallas, TX, USA), for 24 h at 4–5 °C. All antibodies were used at a 1:50 dilution from a 200 μg/mL stock. We mounted coverslips with adherent cells onto microscope slides with 3 μL of DAPI mounting medium. We immediately captured images of the slides using Zeiss M2 epi-fluorescent microscopy (20× objective) with appropriate excitation and emission settings for Alexa Fluor 594 and DAPI. The fluorescence density for each EMT marker was computed in Corel Photo-Paint, with background fluorescence subtracted from the mean fluorescence of the entire image, and the resulting difference multiplied by the pixel density.

### 2.6. Tunneling Nanotube Assay

Cells were seeded onto 12 mm circular glass coverslips placed in sterile 24-well tissue culture plates (Falcon; Becton, Dickinson & Company, Mississauga, ON, Canada) and grown to ~70–80% confluence. The cells were first serum-starved (medium without FBS) for 3 h before incubation with E1S (10 nM). For combined E1S and OP treatment, cells were pretreated with OP (300 μg/mL) for 15 min before adding E1S. Control wells were incubated in serum-free media. Cells were then washed and treated with CellMaskTM Orange Actin Tracking Stain (A57244, Invitrogen, Thermo Fisher Scientific Inc., Waltham, MA, USA) with excitation at 545 nm for 10 min, washed, fixed with 4% PFA, and incubated at 4 °C for 24 h. The following day, cells were washed, and coverslips with attached cells were inverted onto a slide with Vectashield DAPI fluorescent mounting medium (VECTH1500, MJS BioLynx Inc., Brockville, ON, Canada) to stain the nuclei. Slides were immediately visualized using Zeiss M2 epifluorescent microscopy (20× objective magnification; Carl Zeiss Canada Ltd., Toronto, ON, Canada). Cell projections were differentiated from the cell body using Cell Profiler and ImageJ (Version 23.1.0.389). Fluorescence density was further quantified in Corel Photo-Paint X8, Version 24.3.0.57, using the same density equation as previously reported by Baghaie et al. [38].

### 2.7. NF-κB-Dependent Secreted Embryonic Alkaline Phosphatase Assay (SEAP)

Prepare a suspension of 1 × 10^6^ RAW-BlueTM cells/mL. Add 100 μL (~100,000 cells) to each well of a flat-bottom 96-well plate and incubate at 37 °C with 5% CO_2_ for approximately 4 h to allow cell adherence. Negative control wells received 100 μL of growth medium with Zeocin. Positive control wells received lipopolysaccharide (LPS; 5 μg/mL; Sigma-Aldrich, Millipore Sigma Canada Ltd., Oakville, ON, Canada). Estrogen-treated cells received 100 μL of E1S (10 nM). Under inhibitory conditions, cells were first treated with BIM-23127, MMP9i, or OP for 15 min, then stimulated with 50 μL of E1S (10 nM), for a total treatment volume of 100 μL. Thereafter, plates were incubated at 37 °C for 18–24 h. SEAP activity was quantified with QUANTI-Blue^TM^ reagent (InvivoGen) in accordance with the manufacturer’s instructions. First, add 180 μL of the reagent to each well of a new 96-well plate, then add 20 μL of supernatant collected from the RAW-Blue^TM^-treated cells. Plates were then incubated at 37 °C for approximately 4 h to allow sufficient signal development. After incubation, SEAP activity was measured by spectrophotometry (SpectraMax 250, Molecular Devices, Sunnyvale, CA, USA) at 650 nm.

### 2.8. AlamarBlue Viability Assay and Metabolic Activity

E1S-induced viability or cytotoxicity was determined using the AlamarBlue assay, as previously described [39]. Cells were seeded at 20,000 cells per well in flat-bottom 96-well plates. Cells were incubated at 37 °C in 5% CO_2_ for 24 h before treatment with E1S or an untreated vehicle control. Cell viability was measured using an AlamarBlue assay (Invitrogen; Thermo Fisher Scientific, Eugene, OR, USA). Cells were treated with 100 μL of E1S at varying concentrations (0.1–50 nM) diluted in serum-starved medium. Control cells received 100 μL of medium with FBS. Medium with FBS was also added to wells without cells to obtain a blank medium control. Cells were then incubated for 24 h at 37 °C with 5% CO_2_. After incubation, each well received 10 μL of AlamarBlue reagent and was incubated for 4 h. We recorded absorbance at 560 nm (excitation) and 590 nm (emission) using a SpectraMax 250 spectrophotometer. The results were calculated by subtracting the blank media control from the fluorescence and compared with the untreated control. The viability of cells was then ascertained using the following formula:

$$\frac{\left[\left(Absorbance\;of\;treated\;cells\right)-\left(media\;absorbance\right)\right]}{\left[(Absorbance\;of\;control\;cells)-(media\;absorbance)\right]}\;\times 100$$

### 2.9. Statistical Analysis

All statistical analyses were performed using GraphPad Prism (version 10, 2025). All analyses were presented as the mean ± the standard error of the mean (SEM). To analyze any differences between two or more independent experimental groups, a one-way analysis of variance (ANOVA) was used at a 95% confidence level, followed by Fisher’s uncorrected LSD post hoc test. Statistical significance was denoted using asterisks, wherein *p* < 0.05, unless otherwise stated.

## 3. Results

### 3.1. E1S Binding GPER Induces Sialidase Activity

**Figure 1:**
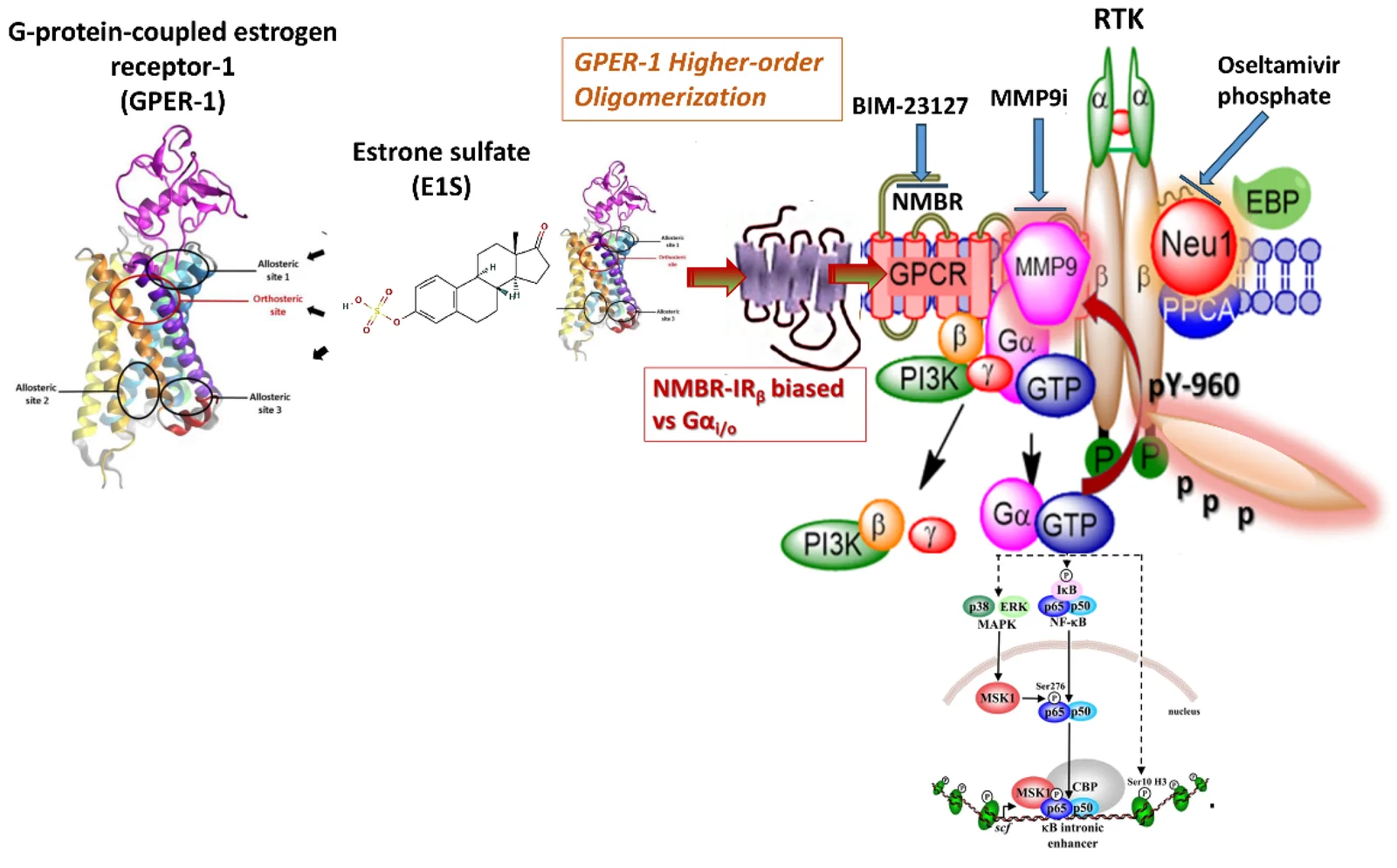
E1S binding to GPER is depicted in the Neu-1-MMP-9-GPCR signaling cross-talk. E1S binding to GPER is illustrated here, with the locations of orthosteric and allosteric binding sites. E1S can bind to orthosteric or allosteric sites. E1S binding to GPER can induce conformational changes in GPER, forming a higher-order oligomerization with GPCR NMBR-MMP-9-Neu-1, which in turn activates Neu-1 by releasing the elastin-binding protein (EBP) from its complex with Neu-1 and the protective protein cathepsin A (PPCA). Activated Neu-1 cleaves α-2,3 sialic acid, removing steric hindrance from the terminal glycosyl residue of the RTK receptor, which in turn induces the E1S-dependent signal from the E1S-binding GPER. Inhibitors of various components of this signaling paradigm are oseltamivir phosphate (OP; inhibitor of Neu-1), MMP9i (MMP-9 inhibitor), and BIM 23127 (NMBR inhibitor). **Abbreviations:** RTK: receptor tyrosine kinase; GPCR: G-protein-coupled receptor; MMP-9: matrix metalloprotease-9; Neu-1: neuraminidase-1; PPCA: protective protein cathepsin A; EBP: elastin-binding protein. Citation: Modified in part from Abdulkhalek et al. [41] (accessed on 7 January 2013), publisher and licensee Dove Medical Press Ltd (London SW1P 1WG, United Kingdom). This is an open-access article that permits unrestricted non-commercial use, provided the original work is properly cited.

A novel cross-talk between neuraminidase-1 (Neu-1) and matrix metalloproteinase-9 (MMP-9) was reported to be in alliance with the GPCR neuromedin B (NMBR), which is essential for ligand-induced receptor tyrosine kinase (RTK) activation and cellular signaling [40]. It remains unclear whether E1S binding to GPER on the cell surface can induce downstream signaling via conformational changes in the signaling paradigm depicted in Figure 1.

**Figure 2:**
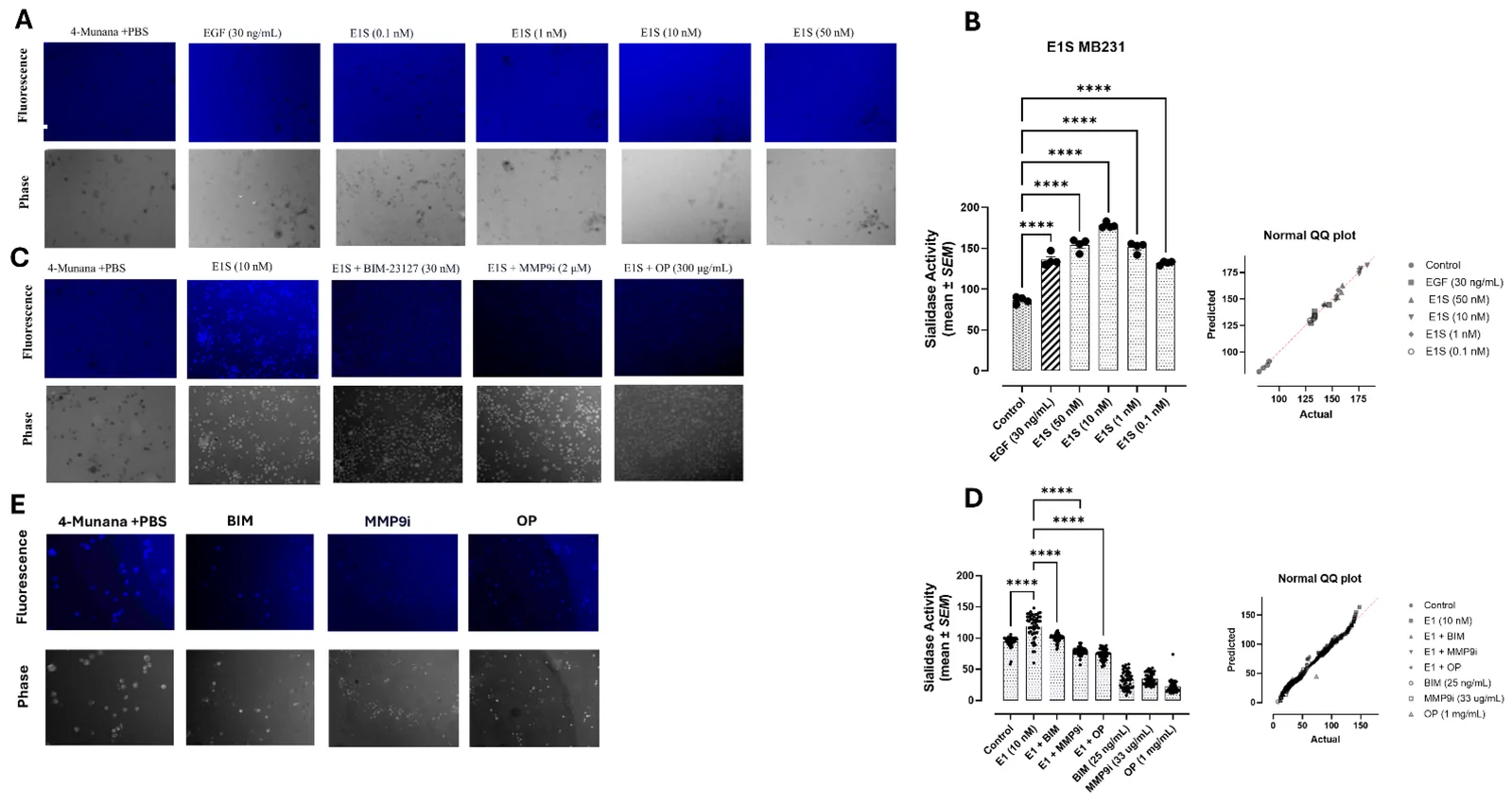
E1S induces Neu1 sialidase activity in MDA-MB-231 triple-negative breast cancer cells. (**A**) After adding 4-MUNANA, MDA-MB-231 cells were treated with EGF as a positive control or with E1S at various concentrations (0.1–50 nM). Fluorescent images were captured using epi-fluorescent microscopy with excitation and emission wavelengths of 365–380 nm and 445–454 nm, respectively, using a 10× objective (scale bar). (**B**) Compared with untreated controls, E1S-treated cells increased fluorescence, suggesting Neu1 sialidase activity. (**C**) E1S (10 nM)-treated cells, together with either BIM23 (30 nM), MMP9i (2 µM), or OP (300 µg/mL), significantly inhibited sialidase activity, as shown in (**D**). Sialidase activity with the three inhibitors alone is shown in (**D**,**E**). A normal QQ plot assesses the dataset normality to verify a normal distribution (**B**,**D**). ImageJ was used to calculate the mean fluorescence at 50 points around the cells’ periphery across four images (*n* = 4). The graphical depiction of the mean ± SEM florescence density demonstrates that E1S upregulates sialidase activity (*p* < 0.0001, *n* = 4), with asterisks signifying statistical significance. Statistical significance among the six independent groups was assessed using ANOVA and Fisher’s uncorrected LSD post hoc test at the 95% confidence level. The data presented were consistent across three independent experiments. The *p* values are indicated above **** astericks.

**Figure 3:**
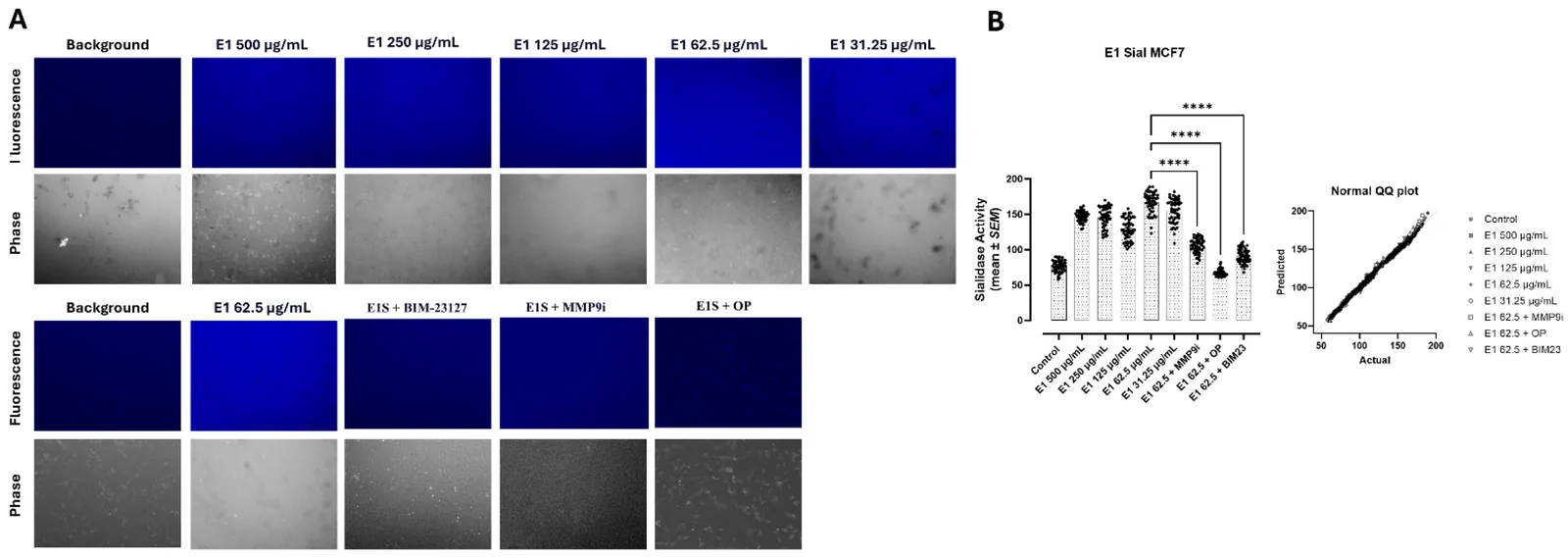
E1S induces Neu1 sialidase activity in MCF-7 breast cancer cells. (**A**) After treatment with 4-MUNANA, MCF-7 cells were exposed to various concentrations of E1S (31.25–500 µg/mL). Fluorescent images were captured using epi-fluorescent microscopy with excitation and emission wavelengths of 365–380 nm and 445–454 nm, respectively, and a 10× objective (scale bar). (**B**) Compared with untreated controls, E1S treatment increased fluorescence, suggesting increased 4-MU and, therefore, increased Neu1 sialidase activity. E1S-treated cells at 62.5 ug/mL together with BIM23 (30 nM), MMP9i (2 µM), and OP (300 µg/mL) significantly inhibited sialidase activity A normal QQ plot assesses whether a dataset follows a normal distribution (**B**). ImageJ was used to calculate the mean florescence at 50 points around the cells’ periphery across four images (*n* = 4). The graph of mean ± SEM florescence density shows that E1S upregulates sialidase activity (*p* < 0.0001, *n* = 4), with asterisks indicating statistical significance. Statistical significance among the five independent groups was assessed using ANOVA and Fisher’s uncorrected LSD post hoc test at 95% confidence. The data presented were consistent across three independent experiments. The *p* values are indicated above **** astericks.

Here, live MDA-MB-231 triple-negative breast cancer (TNBC) cells were treated with epidermal growth factor (EGF, 30 µg/mL) and different concentrations (50, 10, 1, and 0.1 nM) of E1S, or untreated, together with sialidase substrate 4-MUNANA (emission 450 nm, excitation 365 nm) for 1 min (Figure 2A,B). Phase-contrast images showed cells, while fluorescent blue images revealed sialidase activity surrounding the live cells. The data revealed that the control, untreated live cells had negligible sialidase activity compared with the positive control EGF-treated cells and the cells treated with E1S (*p* < 0.0001). E1S-treated cells showed increased sialidase activity, suggesting that sialidase activity results from E1S binding to its GPER and forming a heteromeric complex with the GPCR NMBR-MMP9-Neu-1 signaling platform, as depicted in Figure 1. To test this hypothesis, we investigated whether oseltamivir phosphate (OP), MMP9 (MMP9i), and NMBR (BIM23) would inhibit E1S-induced Neu-1 sialidase activity. The data in Figure 2C,D support the hypothesis that adding OP, MMP9i, and BIM23 to E1S-treated cells significantly blocked E1S-induced sialidase activity. Notably, OP and MMP9i reduced E1S-induced sialidase activity to levels below the untreated control (Figure 2D). The inhibitors alone did not affect cell viability (Figure 2C,D). In addition, all phase-contrast images showed confluent live cells. These findings support the hypothesis that the NMBR-MMP-9-Neu1 signaling platform regulates E1S-induced glycosylated RTK receptors, as depicted in Figure 1. TNBC MDA-MB-231 breast cancer cells lack estrogen, progesterone, and HER2 receptors. MDA-MB-231 cells express the G-protein-coupled estrogen receptor (GPER), a potential therapeutic target [42]. MDA-MB-231 cells are highly aggressive, invasive, and mesenchymal-like. On the other hand, MCF-7 breast cancer cells are sensitive to estrogen (E2), which promotes their proliferation. MCF-7 cells (ER-positive breast cancer) also express the G protein-coupled estrogen receptor (GPER/GPR30), which plays a complex, often paradoxical, role in cancer progression [43]. Here, we investigated whether GPER/GPR30, a GPCR implicated in E1S receptor binding, is involved in the Neu-1-MMP-9-GPCR cross-talk in MCF-7 cells. Live MCF-7 cancer cells were incubated with different concentrations (250, 125, 62.5, and 31.25 µg/mL) of E1S or left untreated, together with the sialidase substrate 4-MUNANA (emission 450 nm, excitation 365nm) for 1 min (Figure 3A). Interestingly, E1S induced sialidase activity in MCF-7 cells, which BIM23, MMP9i, and OP significantly inhibited (Figure 3A,B).

**Figure 4:**
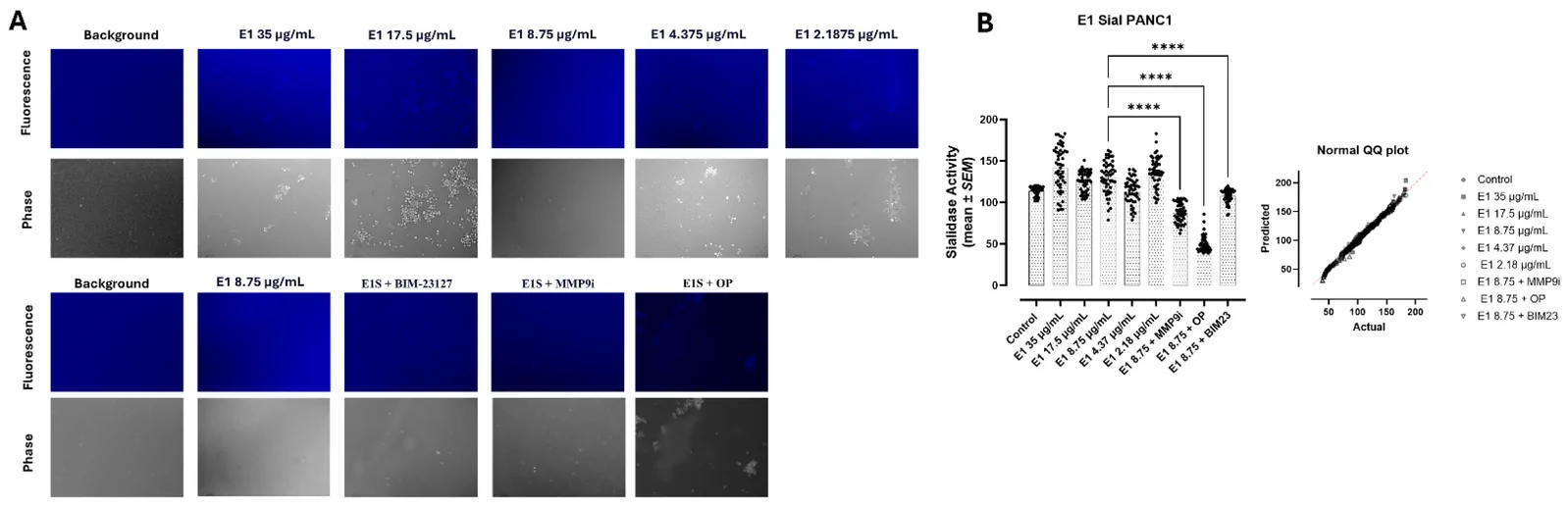
E1S induces Neu1 sialidase activity in PANC-1 pancreatic cancer cells. (**A**) After treatment with 4-MUNANA, PANC-1 cells were exposed to various concentrations of E1S (2.18–35 µg/mL). Fluorescent images were captured using epi-fluorescent microscopy with excitation and emission wavelengths of 365–380 nm and 445–454 nm, respectively, and a 10× objective (scale bar). (**B**) Compared with untreated controls, E1S treatment increased fluorescence, suggesting increased 4-MU and, therefore, Neu1 sialidase activity. E1S-treated cells at 8.75 ug/mL together with BIM23 (30 nM). MMP9i (2 µM) and OP (300 µg/mL) significantly inhibited sialidase activity, as depicted in (**A**,**B**). A normal QQ plot assesses the normality of a dataset, assuming it follows a normal distribution (**B**). ImageJ was used to calculate the mean florescence at 50 points around the cells’ periphery across four images (*n* = 4). The graph of mean ± SEM florescence density shows that E1S upregulates sialidase activity (*p* < 0.0001, *n* = 4), with asterisks indicating statistical significance. Statistical significance among the six independent groups was assessed using ANOVA and Fisher’s uncorrected LSD post hoc test at 95% confidence. The data presented were consistent across three independent experiments. The *p* values are indicated above **** astericks.

Jiang et al. [44] recently reported a disproportionate rise in the incidence of pancreatic cancer in younger women, which was predominantly attributed to pancreatic ductal adenocarcinoma (PDAC) and less to pancreatic neuroendocrine tumors (PanNET). To this end, we investigated the effects of E1S-induced sialidase activity on PANC-1 pancreatic cancer cells, a human pancreatic ductal adenocarcinoma cell line. PANC-1 cells also express both ERα and GPER1, and studies have shown that GPER activation in pancreatic cancer cells, including PANC-1, can inhibit proliferation [45,46]. Here, we investigated whether E1S receptor binding on PANC-1 cells is implicated in the Neu-1-MMP-9-GPCR cross-talk. Live PANC-1 cancer cells were added to different indicated concentrations of E1S or untreated, together with sialidase substrate 4-MUNANA (emission 450 nm, excitation 365nm) for 1 min (Figure 4A). Interestingly, E1S induced sialidase activity in PANC-1 cells, and BIM23, MMP9i, and OP significantly inhibited this activity (Figure 4A,B).

### 3.2. GPER Co-Localizes with Neu1 on the Cell Surface of Naïve Unstimulated MDA-MB-231 and MCF-7, and Less with PANC-1 Cells

**Figure 5:**
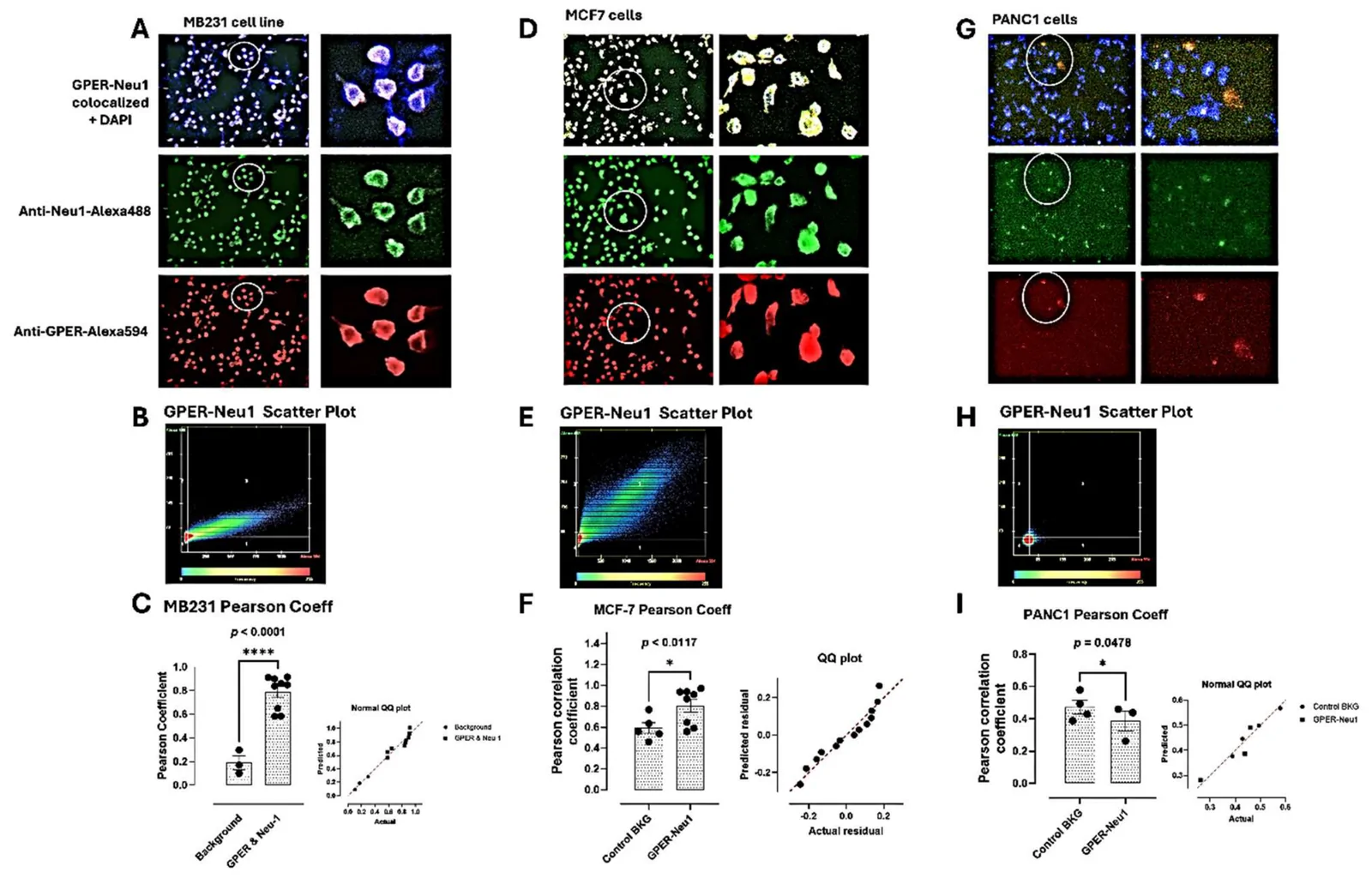
GPER colocalizes with Neu1 in MDA-MB-231 (**A**–**C**), MCF-7 (**D**–**F**), and PANC-1 (**G**–**I**) cancer cells. (**A**,**D**,**G**) show 20× and 40× objective immunofluorescence images of GPER-Neu1 with DAPI. For colocalization analysis, cells were fixed, permeabilized, blocked, and immunostained with anti-GPER conjugated with Alexa Fluor 594 (red) and anti-Neu1 conjugated with Alexa Fluor 488 (green). The circle of cells is enhanced. We quantified colocalization using AxioVision imaging software to compute the Pearson correlation coefficient, which measures the linear association between two variables (20× objective, scale bar). The Pearson correlation coefficient for colocalization between GPER and Neu-1 was 0.7874 in MDA-MB-231 (**C**), 0.8037 in MCF-7 (**F**), and 0.3865 in PANC-1 cells (**I**). A normal QQ plot assesses whether a dataset follows a normal distribution (**C**,**F**,**I**). The Pearson correlation coefficient ranged from 0.8 to 0.9, suggesting a strong association between GPER and Neu1. The graph of mean ± SEM florescence density shows that Pearson Coeff for GPER-Neu-1 compared to control background with asterisks indicating statistical significance of indicated *p* values. Statistical significance among the independent groups was assessed using ANOVA and Fisher’s uncorrected LSD post hoc test at 95% confidence. The *p* values are indicated above * and **** astericks.

Filardo et al. [47] reported that estrogen action requires GPER (GPR30) to release pro-heparan-bound EGF from the cell membrane surface via Gβγ-subunit-dependent transactivation of the epidermal growth factor receptor (EGFR). We have reported that EGFRs are regulated by the molecular organizational signaling platform of Neu1 and MMP-9 cross-talk in alliance with GPCR on the cell surface [48], as well as with neurotrophin factor-induced TrkA [49], insulin [34,36,40,50], and Toll-like (TLR) [51,52] for receptor activation and cellular signaling. Here, we investigated whether GPER forms a complex with the signaling platform of Neu1 and MMP-9, in alliance with the NMBR GPCR, at the cell surface. As depicted in Figure 5, GPERs colocalize with Neu-1 in naïve MDA-MB-231 and MCF-7 breast cancer cells, but to a lesser extent in PANC-1 pancreatic cancer cells. Here, we used the Pearson correlation coefficient, which measures the linear association between two variables. We quantitatively analyzed convergence using the Pearson correlation coefficient, where r = 0.70–0.89 indicates a strong correlation and r = 0.9–1.00 indicates a very strong linear correlation [53]. The Pearson correlation coefficient for GPER-Neu-1 colocalization was 0.7874 for MDA-MB-231 (Figure 5C), 0.8037 for MCF-7 (Figure 5F), and 0.3865 for PANC-1 cells (Figure 5I), affirming GPER-Neu-1 receptor proximity. These findings suggest spatial proximity between GPER and Neu1, supporting their interaction in the proposed signaling platform depicted in Figure 1.

The data in Figure 5 support E1S GPERs involved in the proposed biased GPCR NMBR-MMP9-Neu-1 signaling paradigm depicted in Figure 1. Interestingly, E1S GPER receptors colocalizing with Neu-1 may also be poised to adopt biased active states involving receptor tyrosine kinases (RTKs) and Toll-like receptors (TLRs), with subsequent NF-kB activation. Regulation of these GPER-active states may be mediated by endogenous E1 binding to orthosteric or distinct allosteric sites [15], leading to distinct functional selectivity or ‘biased agonism.’ These results provide novel molecular insights into the intricate heteromer interactions between GPER and other GPCRs in the resting state. This intriguing concept of GPER forming heteromers with different GPCRs underscores biased agonism, particularly through distinct receptor-G protein coupling [15,25].

### 3.3. E1S Significantly Alters Cancer Cell Phenotype to Induce Tunneling Nanotubes (TNTs) in MDA-MB-231 Cells

**Figure 6:**
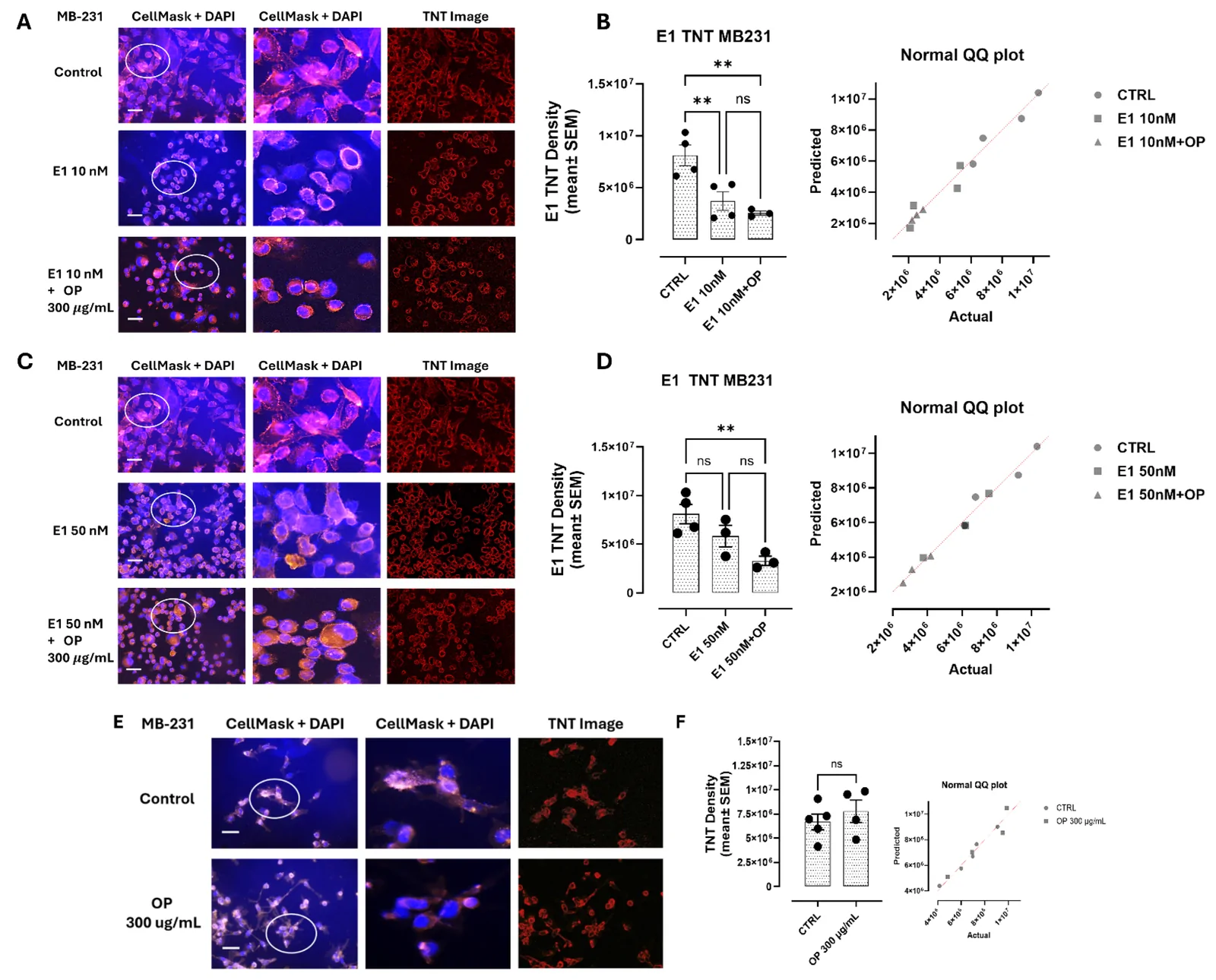
E1S (labeled E1 in image) significantly alters the cancer cell phenotype, inducing the formation of tunneling nanotubes (TNTs) in MDA-MB-231 cells. (**A**,**C**,**E**) Representative CellMask + DAPI and TNT quantification images of cell size and projections following E1S treatment. Slide images were observed using Zeiss M2 epifluorescent microscopy (20× magnification, scale bar), with images captured under the Rhodamine (554 nm) channel. We enhanced the images within the circles (TNT images) and differentiated cell projections in ImageJ. (**B**,**D**,**F**) We quantified the projections using Corel Photo-Paint. (**E**,**F**) Representative TNT images of cells treated with 300 ug/mL of oseltamivir phosphate (OP). A normal QQ plot assesses the normality of a dataset, assuming it follows a normal distribution (**B**,**D**,**F**). TNT quantification for 15 min before E1S treatment. ** *p* < 0.01, ns = not significant.

To assess E1S’s phenotypic impact on cancer cells, we investigated its ability to form tunneling nanotubes (TNTs) in MDA-MB-231 cells at 10 nM after 24 h in culture. We performed an assay using a CellMask membrane stain with an excitation of 554 nm. The results indicate that treating MDA-MB-231 cells with E1S (10nM) significantly decreased the density of cellular projections compared to the untreated control (Figure 6A,B, TNT images). Treating MDA-MB-231 cells with E1S (50nM) resulted in a marked but not significant decrease in the density of cellular projections compared to the untreated control (Figure 6A,B, TNT images). The number of cellular projections can also be visualized in Figure 6C,D (TNT images). Following E1S and treatment with 300 ug/mL of OP, small, thin projections form between neighboring cells and over longer distances, with a significant decrease in TNTs (Figure 6). Additionally, OP treatment alone did not significantly affect the TNT cell phenotype (Figure 6E,F).

### 3.4. E1S Increases Epithelial Markers and Decreases Mesenchymal Markers in MDA-MB-231 and MCF-7 Breast Cancer Cells

**Figure 7:**
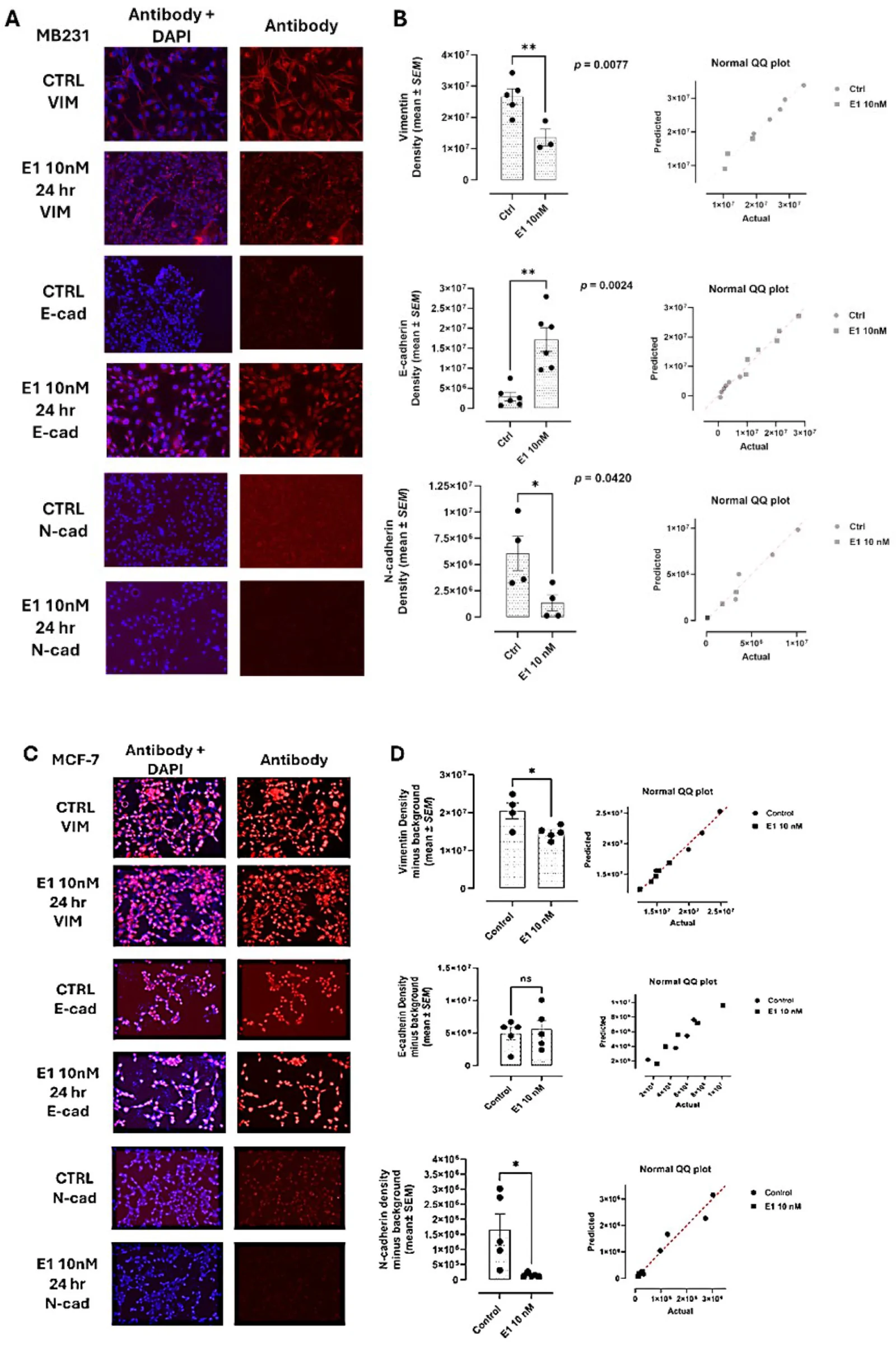
E1S (labeled E1 in image) Increases Epithelial Markers and Decreases Mesenchymal Markers in MDA-MB-231 (**A**,**B**) and MCF-7 (**C**,**D**) Breast Cancer Cells. Cells were treated with primary antibodies conjugated with Alexa Fluor 594 (excitation/emission ~594/~617 nm; appears as red fluorescence) to detect E-cadherin, N-cadherin, or vimentin. Fluorescence imaging using epi-fluorescent microscopy (20× objective, scale bar) shows that E1S treatment (10 nM) increased E-cadherin and decreased N-cadherin and vimentin in MDA-MB-231 and MCF-7 cells. All images were analyzed in Corel Photo-Paint, in which the background fluorescence was subtracted from the mean fluorescence of the entire image, and the resulting difference was multiplied by the pixel density. A normal QQ plot assesses the normality of a dataset, assuming it follows a normal distribution (**B**,**D**). The data presented are representative of three independent experiments. Statistical significance between independent groups was assessed using ANOVA and Fisher’s uncorrected LSD post hoc test at 95% confidence (*p* < 0.05, *n* = 3–6). The *p* values are indicated above * and ** astericks. ns = not significant.

The sialidase and colocalization assays aim to determine whether E1S employs the proposed GPER/NMBR/MMP9/Neu1 signaling axis in MDA-MB-231 and MCF-7 cells. Collectively, the sialidase and colocalization findings suggest that E1S may interact with GPER to induce sialidase activity in a manner dependent on NMBR, MMP9, and Neu1. To assess the effect of GPER/NMBR/MMP/Neu1 signaling on cellular behaviors associated with cancer progression, we performed an epithelial–mesenchymal transition (EMT) assay. As shown in Figure 7A,B, treating MDA-MB-231 cells with E1S (10 nM) for 24 h significantly increased E-cadherin, which is associated with an epithelial, less invasive cellular phenotype. Conversely, E1S (10 nM) treatment for 24 h significantly decreased N-cadherin and vimentin expression, cell markers commonly associated with a mesenchymal, pro-migratory cellular phenotype.

As shown in Figure 7C,D, treatment of MCF-7 cells with E1S (10 nM) for 24 h resulted in a slight, non-significant increase in E-cadherin. Conversely, E1S (10 nM) treatment for 24 h significantly decreased N-cadherin and vimentin expression, cell markers commonly associated with a mesenchymal, pro-migratory cellular phenotype.

### 3.5. E1S Induces Upregulation of the NF-kB Pathway in RAW-Blue^TM^ Reporter Cells

To determine the mechanism(s) of E1S-induced EMT in MDA-MB-231 and MCF-7 breast cancer cells, we investigated its effect on nuclear factor-κB (NF-κB) signaling. Estrone (E1) is the dominant estrogen following menopause, and it has been shown to play a significant role in promoting EMT in ER+ cancers. E1 induces pro-metastatic gene expression, increases inflammatory responses, and promotes the conversion of epithelial cells into mobile, invasive mesenchymal cells [13]. This finding elegantly supports the conceptual framework by proposing that the biased E1 GPER-NMBR-Neu1-MMP9 signaling platform, as depicted in Figure 1, activates NF-kB, which is implicated in the upregulation of EMT markers and metastasis [54].

**Figure 8:**
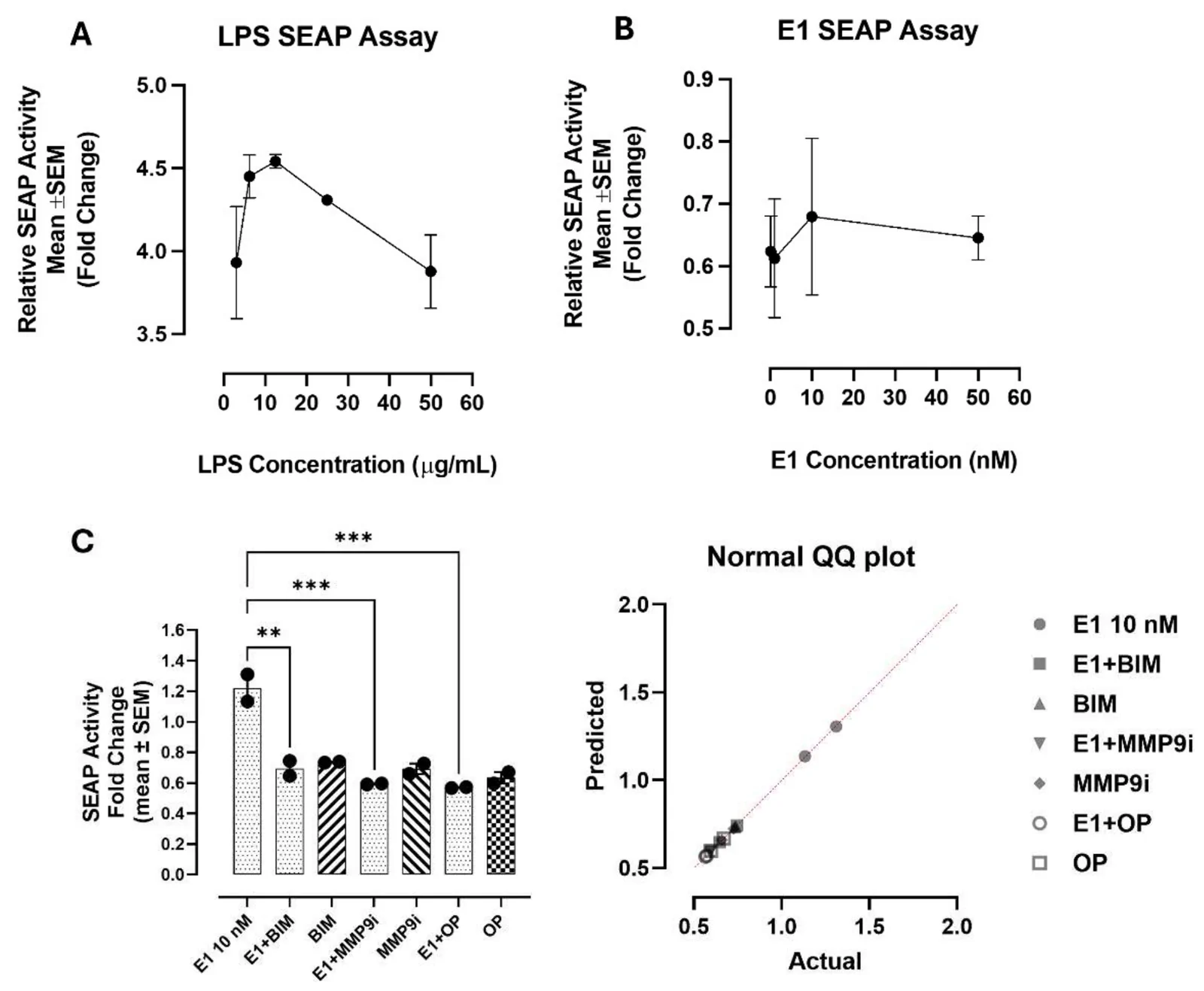
E1S (labelled E1)-treated NF-κB-reporter RawBlue cells revealed NF-κB-induced SEAP expression and secretion, which is inhibited by inhibitors BIM23, MMP9i, and OP. Quantitative spectrophotometric analysis of E1S-induced SEAP activity in the culture medium was performed using QUANTI-Blue^TM^ reagent. The relative SEAP activity was calculated as the fold change for each compound (SEAP activity in medium from treated cells minus background divided by SEAP activity in medium from untreated cells minus background). (**A**) Relative SEAP activity using positive control LPS at different concentrations. (**B**) Relative SEAP activity using E1S (labelled E1) at different concentrations. A normal QQ plot assesses the dataset normality to confirm it follows a normal distribution (**C**). Results are the means of three separate experiments. As indicated by the asterisks, statistical significance was determined using ANOVA and Fisher’s uncorrected LSD post hoc test at a confidence level of 95%. ** *p* < 0.01, *** *p* < 0.001.

To test whether the E1S GPER-NMBR-Neu1-MMP9 signaling platform induces downstream NF-kB activity, we used a mouse macrophage reporter cell line (RAW-Blue™ cells) that stably expresses a secreted embryonic alkaline phosphatase (SEAP) gene inducible by the NF-kB and activator protein-1 (AP-1) transcription factors. AP-1 regulates gene expression in response to various stimuli, such as cytokines, growth factors, stress, and bacterial and viral infections; activated RAW-Blue™ cells secrete SEAP, which is detectable and quantifiable with QUANTI-Blue™, a SEAP detection medium. The data in Figure 8B,C clearly show that E1S-treated RAW-Blue™ cells induced SEAP secretion, which was inhibited by BIM-23, MMP-9i, and OP, and therefore reduced NF-kB activity compared with E1S alone. To confirm that RawBlue cells have an active SEAP reporter, lipopolysaccharide (LPS)-treated cells induced SEAP activity (Figure 8A).

Qureshi et al. [13] have reported that estrone (E1), the primary estrogen in postmenopausal women, drives ER+ breast cancer metastasis by promoting EMT. Produced largely in adipose tissue, E1 stimulates cancer cell invasion. It acts as a pro-inflammatory agent to accelerate metastasis, particularly in obese, postmenopausal individuals. Bergers and Fendt [55] reported that metastasizing cancer cells can surreptitiously adapt their metabolic activity during their metastatic invasion. Gorski et al. [56] reported that cyclin E1 affects cancer metabolism and is significantly associated with chemoresistance. By initiating invasion-related communications, cancer cells can rewire their metabolism to promote proliferation and migration and to counter metabolic stress during progression [57]. During this reprogramming process, cancer cells’ metabolism and other cellular activities are integrated and mutually regulated by tunneling nanotube (TNT) communication, thereby altering specific metabolic drivers of tumor growth and progression [25,57].

**Figure 9:**
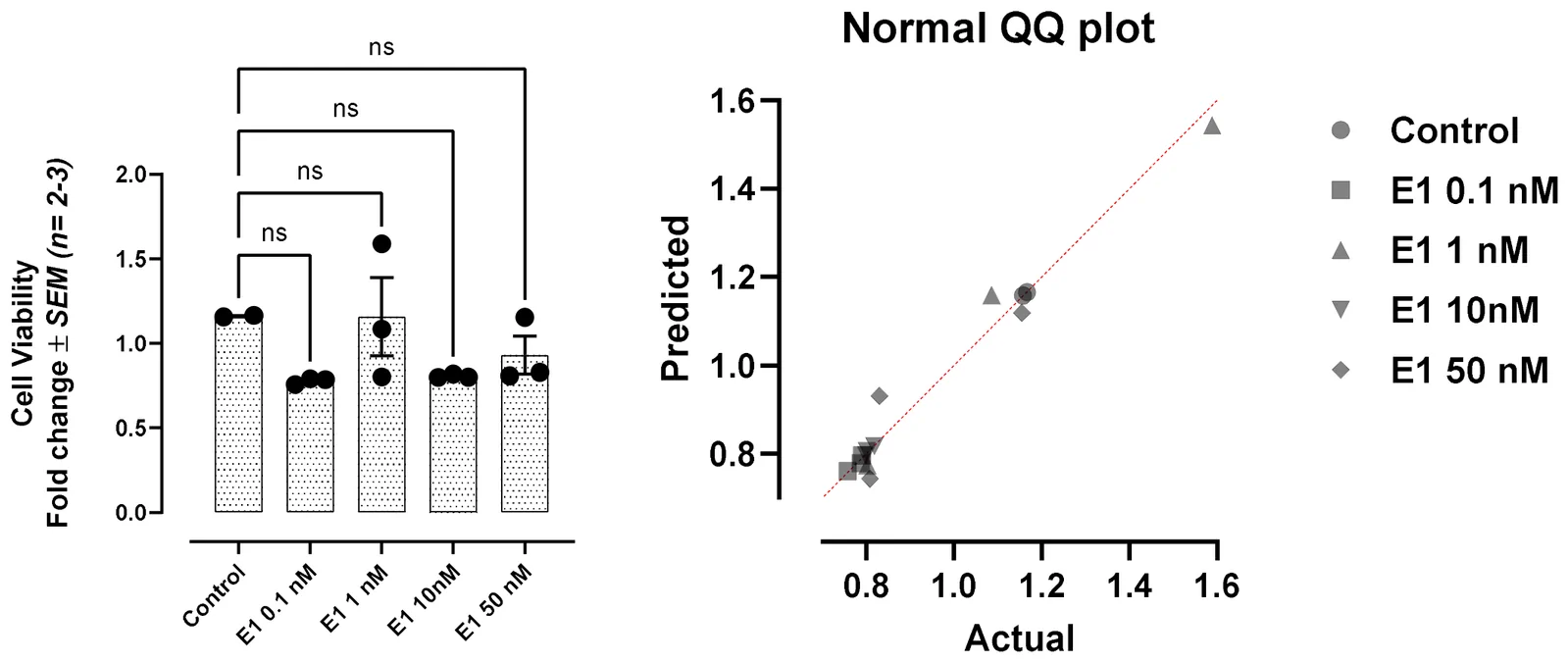
E1S (labelled E1) treatment did not affect cell viability and metabolic activity in MDA-MB-231 cells using the AlamarBlue assay at different E1S doses. Cells were plated at ~20,000 cells per well in a flat-bottom 96-well plate containing conditioned culture medium for 24 h at 37 °C and 5% CO_2_. Following incubation, the cells were treated with 90 μL of each E1S at the indicated doses in FBS-free medium for 15 min before E1S stimulation. Controls had medium with FBS only. We added 10 μL of AlamarBlue reagent to each well and incubated the mixture for 4 h. We measured absorbance at 570 nm using a spectrophotometer. Data are represented as the mean ± SEM of 3 independent experiments performed in triplicate. Cell viability was determined as the fluorescence corrected for the blank media control divided by the untreated cellular control. A normal QQ plot assesses the normality of a dataset, assuming it follows a normal distribution. Statistical significance was calculated with ANOVA and Fisher’s uncorrected LSD post hoc test at a confidence level of 95%. ns = non-significant.

To this end, we investigated the metabolic activity of MDA-MB-231 cells following E1S treatment. E1S treatment did not affect cell viability or metabolic activity in MDA-MB-231 cells, as assessed by the AlamarBlue assay at different E1S doses (Figure 9). Additionally, cell viability remained at 80–95% with E1S treatment, similar to the untreated control, indicating that E1S did not cause nonspecific cell death.

### 3.6. E1S Inhibited the Migratory Potential of Breast MDA-MB-231, MCF-7, and Pancreatic PANC-1 Cancer Cells in a Scratch Wound Assay

**Figure 10:**
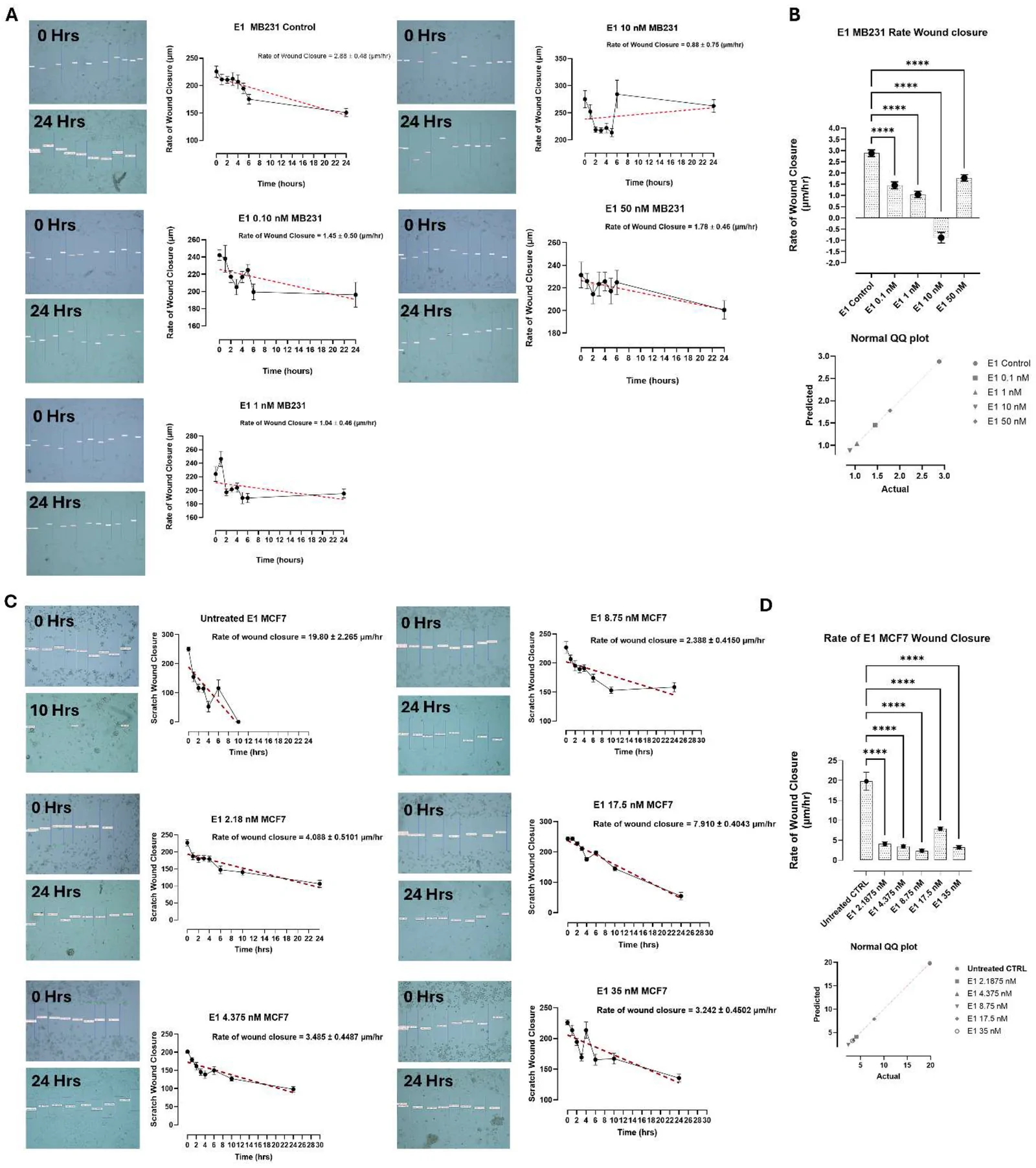

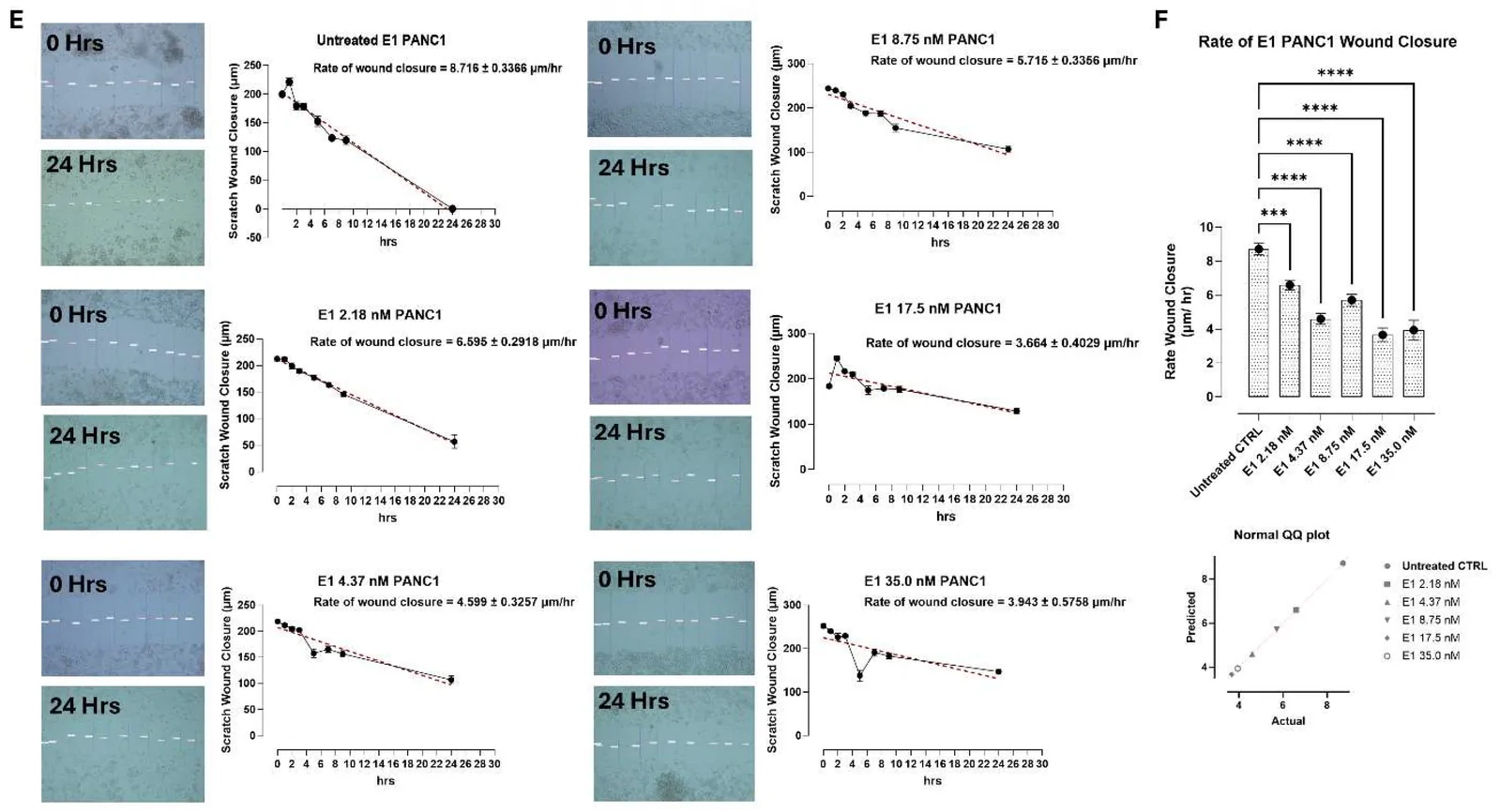
E1S ablates the migratory potential of (**A**,**B**) MDA-MB-231, (**C**,**D**) MCF-7, and (**E**,**F**) pancreatic PANC-1 cancer cells in a scratch wound assay. Cells were cultured in a 50 mm glass bottom MatTek culture dish and allowed to adhere to 90% confluence on the glass slide in an incubator at 37 °C and 5% CO_2_. A sterile pipette tip created a scratch wound, and non-adherent cells were removed after washing. The control cells were supplemented with a medium containing 5% fetal bovine serum (FBS). In contrast, the treated cells were supplemented with E1S at the indicated dosages. We imaged cells with a Nikon Eclipse Ti2 microscope (4× magnification, scale bar) every hour for the first 6 h and at 24 h after scratch creation. We measured wound width at 6–8 points per image using NIS-Elements AR software (version 5.21.00), and analyzed the results using simple linear regression (red dashed line). We used GraphPad Prism 10 to measure the rate of wound closure, expressed as μm/h. The slope of the linear regression line is the rate of wound gap closure in μm/h ± standard error of the slope. (**B**,**D**,**F**) Normal QQ plots assess whether a dataset for rate of wound closure follows a normal distribution. The quantified data represent two to three independent experiments displaying similar results. Statistical significance, as indicated by asterisks, was calculated with ANOVA and Fisher’s LSD uncorrected multiple comparisons at a confidence level of 95%. **** *p* < 0.0001 and *** *p* < 0.001.

The data in Figure 7 clearly demonstrate that E1S significantly reduced the expression of the invasive EMT markers N-cadherin and vimentin in MDA-MB-231 cells after 24 h of exposure. These observations suggest that E1S may also affect the migratory potential of these cancer cells. Figure 10 shows the migratory rate of E1S from the scratch wound area over 24 h. The untreated MDA-MB-231 control cells showed near-complete wound closure at 2.88 ± 0.48 μm/h within 24 h (Figure 10A,B). In contrast, E1S treatment significantly inhibited wound closure in MDA-MB-231 cells at 0.1, 1, 10, and 50 nM, with inhibition occurring within 24 h. These data suggest that E1S significantly inhibited MDA-MB-231 cell migration, potentially by abrogating EMT-mediated metastasis, as shown in Figure 7.

Similarly, the untreated MCF-7 (Figure 10C,D) control cells showed near-complete wound closure at 19.80 ± 2.26 μm/h within 24 h (Figure 10C). Interestingly, wound closure in E1S-treated MCF-7 cells at the indicated dosages was significantly inhibited compared with untreated control cells, with all effects occurring within 24 h (Figure 10D). Similarly, the wound closure rate for E1S-treated PANC-1 cells (Figure 10E,F) at the indicated dosages was significantly inhibited compared to untreated control cells, with all effects occurring within 24 h (Figure 10F). These data suggest that E1S-treated cells significantly inhibited the migration and potential invasiveness of MDA-MB-231 and MCF-7 breast cancer cells and PANC-1 pancreatic cancer cells, which had already undergone EMT.

## 4. Discussion

Fuentes and Silveyra [58] reviewed the molecular events governing gene expression regulation via the nuclear estrogen receptors (ERα and ERβ) and the membrane estrogen receptor (GPER1). Estrogen can activate intracellular signaling cascades by interacting with GPER1 and/or ERα and ERβ. Estrogen-mediated signaling events can be divided into genomic and non-genomic events. Genomic effects involve the migration of estrogen receptor complexes into the cell nucleus. In contrast, non-genomic effects involve indirect regulation of gene expression via various intracellular signaling pathways. More recently, the non-genomic effects of estrogen involve a new type of estrogen-binding protein, the G protein-coupled estrogen receptor-1 (GPER1), or membrane estrogen receptor [58]. In addition, Martinotti [59] proposed a possible biological connection between GPER1-mediated non-genomic estrogen signaling and telomerase. The non-genomic effects of estrogen E1S may affect estrogen availability by inducing GPER1 and G proteins, regulating telomerase reverse transcriptase (hTERT), and maintaining telomeres. A telomere is a region of repetitive sequences at each end of the chromosomes of most eukaryotes. Telomeres protect chromosome ends from DNA damage or fusion with neighboring chromosomes.

GPCRs comprise the largest family of cell-surface signal-transduction molecules in mammalian cells. They have long been implicated in transactivating RTKs in the absence of their respective growth factors, particularly receptors that bind epidermal growth factor, platelet-derived growth factors, fibroblast growth factor, and neurotrophins [60]. Delcourt and colleagues provide an eloquent review of the molecular mechanisms underlying this novel cross-communication between GPCRs and RTKs [61]. Reciprocally, growth factor signaling through RTKs also utilizes GPCR signaling molecules to initiate the molecular signaling platform underlying a novel Neu1-MMP-9 cross-talk in alliance with RTK on the cell surface. We identified this novel GPCR-signaling platform as critically essential for neurotrophin factor-induced TrkA [49], insulin [34,50], and Toll-like receptor (TLR) [51,52] activation and cellular signaling. These findings have revealed a novel concept in which GPCR activation is essential for growth-factor RTK and pathogen-sensing TLR activation. The mechanism(s) here involves the formation of a functional signaling complex between the GPCR-RTK and GPCR-TLR partners.

Here, we provide novel evidence that estrone (E1S) significantly and dose-dependently induces sialidase activity in live MDA-MB-231, MCF-7, and PANC-1 cancer cells in vitro (Figure 2, Figure 3 and Figure 4). To support our hypothesis that E1S induces Neu-1 specifically, we used three Neu-1 inhibitors, BIM-23127, MMP-9i, and OP, and found that sialidase activity was blocked. Moreover, these findings support our proposed signaling paradigm (Figure 1), in which E1S-binding GPER induces cross-talk between NMBR, MMP9, and Neu-1 sialidase. To confirm these results, a colocalization assay was used to determine E1S GPER’s proximity to Neu-1 on the cell surface. The results firmly established that E1S GPER and Neu-1 form a complex, supporting our hypothesis. The colocalization assay results indicate strong-to-very strong colocalization between GPER and Neu1. By triangulating the close spatial proximity between GPER and Neu1 observed in the colocalization assay with the pathway dependence observed in inhibitor-sensitive sialidase assays, the functional interaction between GPER and Neu1 is presumed in the NMBR/MMP9/Neu1 signaling paradigm [62]. However, the physical interaction between GPER and Neu1 can only be inferred from colocalization. Additional assays, such as co-immunoprecipitation, are needed to confirm this. Alternatively, bioluminescence resonance energy transfer (BRET) can visualize protein interactions in living cells [63]. BRET imaging relies on a luminescent donor that transfers energy to an acceptor molecule, the fluorescence acceptor, under permissive distances (<10 nm) and proper orientations. Moreover, given the contentious localization of GPER, a time-course analysis with additional cell-type markers may yield intriguing and valuable results [33].

Previous studies have highlighted Neu-1’s role in tumorigenesis and metastasis [40]. We hypothesized that E1S affects the expression of EMT markers, including E-cadherin, vimentin, and N-cadherin, involving the NMBR-MMP9-Neu1 signaling paradigm. Figure 7 clearly shows that E1S increases the epithelial marker E-cadherin and decreases the mesenchymal markers vimentin and N-cadherin in MDA-MB-231 and MCF-7 breast cancer cells. Currently, the literature on GPER suggests that its functional consequences in TNBC are highly context-dependent and variable [23,64]. Some studies report that GPER is involved in pro-cancerous pathways. In contrast, other groups report that activation may inhibit breast cancer proliferation [23,64]. Importantly, the pro-adhesive phenotype observed does not contradict previous findings, given the novelty of the present research and the potential E1S agonism of GPER in breast cancer cells.

To further elaborate on the EMT assay findings, we found that E1S shifted the pro-migratory phenotype of MDA-MB-231 cells toward a more epithelial, less invasive phenotype by upregulating E-cadherin and downregulating N-cadherin and vimentin (Figure 10). EMT typically involves a loss of adhesion molecules, such as E-cadherin, and a concomitant upregulation of mesenchymal markers, including N-cadherin and vimentin [65]. In particular, elevated vimentin expression is associated with aggressive phenotypes and poorer prognoses in invasive breast cancer, especially in TNBC [65,66]. However, E-cadherin has also been reported to play a paradoxical role [65]. It is generally assumed that E-cadherin is uniformly lost during breast cancer invasion; however, histological findings indicate that several solid tumor types retain E-cadherin expression, and most metastatic carcinomas collectively invade from the primary tumor [65]. For instance, a retrospective observational study found that high E-cadherin and vimentin expression were associated with decreased recurrence-free survival (RFS) and overall survival (OS) [65]. Therefore, hybrid EMT states are associated with particularly invasive cancers [65,66]. This observation may be attributable to the specific combinatorial expression pattern of EMT markers, which influences the extent of cell migration and metastasis [65,66]. Notably, in this study, vimentin expression decreased alongside N-cadherin, whereas E-cadherin expression increased with E1S treatment at different dosages. This expression profile may be associated with the decreased cell migration we observed in scratch wound assays following E1S treatment (Figure 10).

Nevertheless, when interpreting EMT and scratch wound data, we must consider our consistent use of 2D cell culture models, which do not fully replicate the in vivo environment [67]. For instance, 2D models exhibit altered cell polarity and phenotypic diversity that do not mimic the natural tumor architecture [67]. As such, further experiments in 3D culture models and animal models, despite potential challenges with reproducibility and interpretation, may improve the translational potential of the findings to in vivo environments [67].

Another phenotypic alteration observed in the present study was decreased tunneling nanotube formation in response to E1S stimulation. This lack of tunneling nanotube formation may correlate with a more epithelial, less invasive cellular phenotype. However, a direct causal relationship between tunneling nanotube formation and EMT marker expression cannot be established unless EMT markers are monitored in parallel with direct inhibition of tunneling nanotube formation, such as through pharmacological targeting of the tunneling nanotube F-actin core [68,69].

More broadly, the observed increases in E-cadherin expression, alongside reductions in N-cadherin, vimentin, and tunneling nanotube expression, may conventionally be associated with attenuation of NF-κB- and AP-1-dependent transcriptional mechanisms. However, E1S stimulation was associated with increased SEAP activity, suggestive of increased NF-κB/AP-1 transcription. Here, E1S may elicit a temporally constrained or context-dependent NF-κB/AP-1 transcriptional response, possibly mediated by rapid, non-genomic GPER signaling. This transient, biased E1S-mediated NF-κB/AP-1 activation may support specific transcriptional patterns and the co-activation of alternative pathways, enabling limited proliferative and survival programs without attaining the signaling amplitude or duration required to drive EMT and invasion. It is noteworthy that Kaszycki and Kim [70] and others [71] provided an extensive review on the role of the NLRP3 inflammasome and NF-κB signaling pathways, noting that genetic factors as well as epigenetic modifications are regulated by NF-κB, including DNA methylation, histone modifications, and ncRNA involvement, all of which contribute to the control of gene expression. Notably, data in Figure 7C,D show that treating both MDA-MB-231 and MCF-7 cells with E1S (10 nM) for 24 h resulted in a slight or marked increase in E-cadherin. Conversely, E1S (10 nM) treatment for 24 h significantly decreased N-cadherin and vimentin expression, cell markers commonly associated with a mesenchymal, pro-migratory cellular phenotype.

Interestingly, E1S and the G protein GPER1 (GPR30) are related to G-protein signaling and neuroinflammation. M. Felaco and P. Felaco [72] reported that G-protein signaling involves the transduction of neurotransmitters such as neurotensin, exerting their effects in the nervous system. GPCRs can translate neuropeptide neurotensin binding into intracellular responses that regulate neuronal excitability, synaptic transmission, and gene expression. Neurotensin and neuromedin N are related neuropeptides that share structural homology, particularly in their C-terminal regions, which are critical for biological activity and receptor binding. Both peptides bind to neurotensin receptors (NTS1 and NTR3). However, neurotensin has a higher affinity and potency than neuromedin N, leading to distinct physiological effects. It acts through neurotensin type 2 (NTS2) GPCR receptors, which mediate its effects. Neuromedin N and B are both neuropeptides with distinct roles and functions in the body. Neuromedin B, on the other hand, is a bombesin-related peptide found in the central nervous system and gastrointestinal tract. It regulates various functions by binding to its high-affinity cell-surface receptor, the neuromedin B GPCR (NMBR).

Postmenopausal women are at a higher risk of neuroinflammation, which can lead to cognitive decline and neurodegenerative diseases. The decline in estrogen during perimenopause and menopause is a significant factor in this risk. Estrogen plays a crucial role in regulating various neural processes, including synaptic plasticity, cerebral glucose metabolism, mitochondrial function, neuroinflammatory signaling, and cerebrovascular regulation. Understanding the interplay between aging, hormonal changes, nutrition, and neuroinflammatory processes is essential for developing effective preventive strategies to preserve cognitive function and reduce the risk of neurodegenerative diseases in women.

Interestingly, Figure 1 shows that E1S binding to GPER induces conformational changes in GPER, forming a higher-order oligomer with GPCR NMBR to activate the MMP-9-Neu-1 signaling pathway. Indeed, Alghamdi et al. [50] and Haxho et al. [34,40] found that the GPCR NMBR exists in a protein complex with insulin IRβ subunits on the cell membrane. They showed that IRβ subunits colocalize with NMBR, in complex with mammalian Neu1 and MMP9, in a rat hepatoma cell line (HTC-WT) and in HTC-IR cells that overexpress human insulin receptors, independent of insulin stimulation.

Metastasizing cancer cells can surreptitiously adapt their metabolic activity during their invasion by inducing EMT markers. Cancer cells can initiate communication pathways that facilitate invasion and reprogram cellular activities. During this reprogramming process, cancer cells’ metabolism and other cellular activities are integrated and mutually regulated through tunneling nanotube communication, thereby altering specific metabolic drivers of tumor growth and progression. Cell viability is a critical aspect of cancer progression. Understanding cell viability is crucial in cancer research, as it affects cell proliferation, growth, and metastasis, and helps determine the efficacy of potential therapeutics [28,31]. The AlamarBlue assay found that E1S did not affect cell viability in the MDA-MB-231 cell line (Figure 9). Notably, BIM-23, MMP-9i, and OP did not affect cell viability compared with untreated controls. These findings are noteworthy because they indicate that E1 did not affect cancer cell viability. Moreover, it indicates that E1S does not affect viability or metabolic functional drivers but blocks invasion-related communication by activating Neu-1.

Altogether, these findings support a novel model in which E1S engages rapid, non-genomic, functionally biased GPER signaling to promote transient NF-κB and AP-1 activity. This constrained response in amplitude and duration is consistent with the observed decrease in tunneling nanotubes, the upregulation of epithelial markers, and the suppression of mesenchymal markers. Therefore, E1S-induced GPER activation may enable precise control of NF-κB and AP-1 activity, which fail to match the sustained signaling threshold required to drive EMT, cancer cell migration, and tunneling nanotube formation. These observations highlight the importance of acknowledging dynamic, temporal signaling mechanisms in determining downstream cellular outcomes.

## 5. Limitations

While the study provides novel insights into the role of E1S-mediated signaling in sialidase activity, EMT phenotypes, tunneling nanotube behavior, and SEAP activity, several limitations warrant consideration. For instance, the study used only E1S as an agonist. To better reflect GPER’s physiological relevance, the authors should use a broader range of agonists. Furthermore, although breast and pancreatic cancer cells were used, this study did not use any GPER antagonists. Similarly, using 3D culture systems and in vivo models may more accurately recapitulate the complex interplay of factors present in the TME.

## 6. Conclusions

The role of ovarian hormones in modulating the progression of breast cancers has been reported since 1895 [73]. However, the role of weaker estrogens, such as E1 in its predominant storage form, E1S, and their respective signaling pathways has been understudied. Using sialidase and colocalization assays, the present study found that E1S promotes biased GPER signaling via the NMBR/MMP9/Neu1 pathway in MDA-MB-231, MCF-7, and PANC-1 cells. In the TNBC cell line, E1S treatment increased E-cadherin and decreased N-cadherin and vimentin. Exposure to E1S also decreased the density of tunneling nanotubes, suggesting reduced intercellular communication and reduced transmission of materials conducive to metastasis.

However, despite the observed shift toward a more epithelial phenotype and reduced tunneling nanotube projections, SEAP activity increased. This apparently paradoxical increase in SEAP activity and concomitant reduction in pro-migratory characteristics may be explained through temporal, dynamic signaling wherein the weak, conjugated E1 hormone, E1S, transiently activates NF-κB and AP-1 through the fast-acting GPER in a manner that is too modest in duration and magnitude to drive EMT, cellular migration, and intercellular communication. Overall, these findings suggest that signaling through GPER and NMBR/MMP9/Neu1 is biased, with effects on cellular behavior depending on the strength and duration of the activating ligand. This biased signaling may be leveraged to treat breast and pancreatic cancers, wherein ligands of varying strengths, such as E1S, and their downstream signaling mechanisms can be investigated and exploited to mitigate cancer progression.

## Data Availability

All data needed to evaluate the paper’s conclusions are present. The preclinical data sets generated and analyzed during the current study are not publicly available. However, they can be obtained from the corresponding author upon reasonable request. The data will be provided after review and approval of a research proposal and Statistical Analysis Plan, and execution of a Data Sharing Agreement. The data will be accessible for 12 months for approved requests, subject to possible extensions; contact szewczuk@queensu.ca for more information on the process or to submit a request.

## Abbreviations

4-MU	4-methylumbelliferone
4-MUNANA	2-(4-methylumbelliferyl)-α-D-N-acetylneuraminic acid
AC	Adenylyl cyclase
Akt	Protein kinase B
ANOVA	One-way analysis of variance
AP-1	Activator protein-1
Bcl-2	B-cell lymphoma 2
BRCA1	Breast cancer type 1 susceptibility gene
BRCA2	Breast cancer type 2 susceptibility gene
BRET	Bioluminescence-based resonance energy transfer
BSA	Bovine serum albumin
cAMP	Cyclic adenosine monophosphate
E-cadherin	Epithelial cadherin
E1	Estrone
E1S	Estrone sulfate
E2	Estradiol
E3	Estriol
E4	Estetrol
EBP	Elastin-binding protein
EGF	Epidermal growth factor
EMT-ATFs	EMT-activating transcription factors
EMT	Epithelial-to-mesenchymal
ER	Estrogen receptor
ERK1/2	Extracellular signal-regulated kinases 1/2
ERα	Estrogen receptor α
ERβ	Estrogen receptor β
EZH2	Enhancer of Zeste Homolog 2
GPCR	G protein-coupled receptor
GPER	G protein-coupled estrogen receptor
GPR30	Orphan G protein-coupled receptor
GRK	G protein-coupled receptor kinase
HB-EGF	Heparin-binding epidermal growth factor
EGFR	Human epidermal growth factor receptor
HER2	Human epidermal growth factor receptor 2
HSD17B1	17β-hydroxysteroid dehydrogenases 1
HSD17B2	17β-hydroxysteroid dehydrogenases 2
IGF-1	Insulin-like growth factor-1
IGF-R1	Insulin-like growth factor receptor-1
IKK	IκB kinase
IL-8	Interleukin-8
IM	immunomodulatory
IR	Insulin receptor
IRα	Insulin receptor α
IκB	Inhibitor of κB
LBD	Ligand binding domain
LPA	Lysophosphatidic acid
LPS	Lipopolysaccharide
MAPK	Mitogen-activated protein kinase
MMP	Matrix metalloproteinase
MMP9	Matrix metalloproteinase 9 MMP9i: MMP9 inhibitor
MSL	Mesenchymal stem-like
mTOR	Mammalian target of rapamycin
N-cadherin	Neural cadherin
Neu1	Neuraminidase 1
NF-κB	Nuclear factor-κB
NMB	Neuromedin
NMBR	Neuromedin B receptor
OP	Oseltamivir phosphate
OS	Overall survival
PBS	Phosphate-buffered saline
PD-1	Programmed cell death protein
PD-L1	Programmed death-ligand 1
PDGF	Platelet-derived growth factor
PFA	Paraformaldehyde
PI3K	Phosphoinositide 3-kinase
PIK3CA	Phosphatidylinositol 4,5-bisphosphate 3-kinase catalytic subunit alpha
PIP2	Phosphatidylinositol 4,5-bisphosphate
PIP3	Phosphatidylinositol 3,4,5-trisphosphate
PKA	Protein kinase A
PKC	Protein kinase C
PPCA	Protective protein cathepsin A
PR	Progesterone receptor
RB1	Retinoblastoma
RKIP	Raf-1 kinase protein inhibitor
RTK	Receptor tyrosine kinase
SEAP	Secreted embryonic alkaline phosphatase
SG	Sacituzumab govitecan
STS	Estrogen sulfatase
TBS	Tris-buffered saline
TBST	Tween-20 in Tris-buffered saline
TGF-β	Transforming growth factor-β
TLR	Toll-like receptor
TNBC	Triple-negative breast cancer
TNF-α	Tumor necrosis factor-α
TP53	Tumor protein p53
TrkA	Tropomyosin receptor kinase A
VEGF	Vascular endothelial growth factor
ZEB1/2	Zinc finger E-box binding homeobox 1/2
